# Smart mesoporous silica nanoparticles for precision cancer therapy: Tumor targeting and stimuli‐responsive controlled release

**DOI:** 10.1002/smo2.70103

**Published:** 2026-09-24

**Authors:** Majdi Al‐Amili, Lei Lei, Zhu Jin

**Affiliations:** ^1^ Department of Pharmacology Shantou University Medical College Shantou Guangdong China; ^2^ Shanghai Frontiers Science Center of Drug Target Identification and Delivery School of Pharmaceutical Sciences Shanghai Jiao Tong University Shanghai China; ^3^ Qujing College of Medicine & Health Sciences Qujing China

**Keywords:** controlled release, mesoporous silica nanoparticles, precision cancer therapy, smart molecules, stimuli‐responsive, tumor targeting

## Abstract

Mesoporous silica nanoparticles (MSNs) are useful carriers for precision cancer therapy because their ordered pores, large surface area, controllable particle morphology/structure, and modifiable surface support high cargo loading, tumor targeting, and controlled release. Their role is no longer limited to passive drug storage. By adding molecular gatekeepers, cleavable linkers, responsive polymers, ligands, magnetic components, photosensitizers, or imaging modules, MSNs can translate tumor‐related microenvironment signals or externally applied energy into site‐specific therapeutic activation. This review discusses stimuli‐responsive MSNs from the viewpoint of molecular design and release control. We first examine how shape, size, pore architecture, and surface functionalization influence transport, cellular uptake, loading, and release. We then summarize passive and active targeting strategies, followed by intrinsic stimuli‐responsive systems, including pH‐, enzyme‐, redox‐, reactive oxygen species (ROS)‐, hypoxia‐, glucose‐, ATP‐, ionic‐strength‐, and urea‐responsive MSNs, and extrinsic systems triggered by light, magnetic fields, ultrasound, temperature, and electric fields. Multi‐stimuli‐responsive and combination systems are discussed as examples of how tumor cues, external activation, imaging, and therapy can be coordinated. The final section considers disease‐oriented design, cancer immunotherapy, degradable silica frameworks, long‐term safety, reproducible manufacturing, and clinical translation. Together, these topics show how MSNs can move from porous drug reservoirs toward functional nanoplatforms for precision oncology.

## Graphical abstract

Smart mesoporous silica nanoparticles integrate tunable pore structures, molecular gates, targeting ligands, and stimuli‐responsive components to achieve controlled drug release for precision cancer therapy. This review highlights design principles, therapeutic applications, and future directions in immunotherapy, biodegradability, biosafety, and translation.

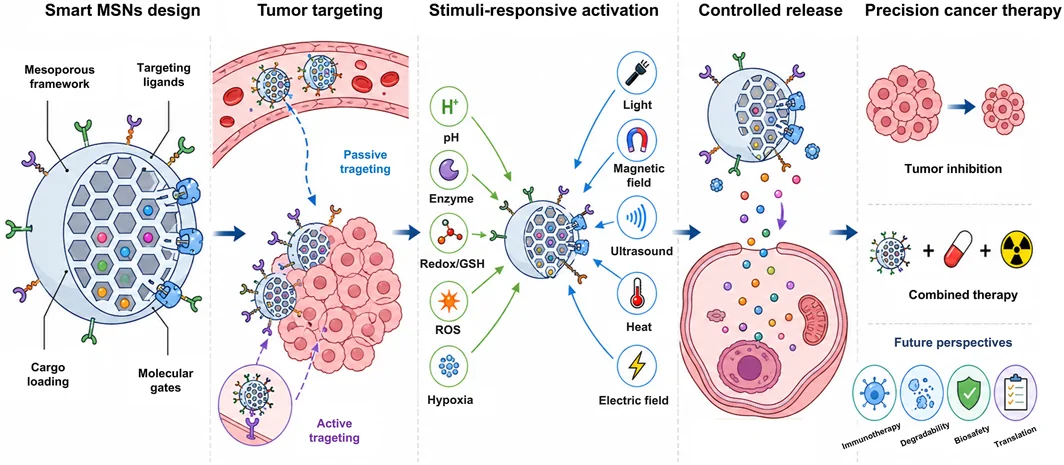

## 1 INTRODUCTION

Nanomedicine constitutes a significant approach in cancer therapy, facilitating targeted and controlled drug delivery. It enhances pharmacokinetics, diminishes systemic toxicity, and aids in the clinical translation of chemotherapy, phototherapy, nucleic acid therapy, and immunotherapy [1]. Clinically translated cancer nanomedicines mainly improve therapeutic indices by altering pharmacokinetics and biodistribution, and newer systems increasingly include actively targeted, stimuli‐responsive, combination‐therapy, and immunotherapy‐oriented formulations [2].

A wide array of nanosized drug delivery systems has been designed with varying sizes, architectures, and surface properties. These drug delivery systems include liposomes, polymeric micelles, nanocrystals, metallic nanoparticles, solid lipid nanoparticles, nanoemulsions, dendrimers, carbon nanotubes, nanogels, and nanocapsules [3, 4, 5]. They can effectively improve drug delivery efficiency and treatment efficacy by avoiding drug degradation by environmental factors, improving pharmacokinetics, increasing drug solubility, and controlling drug release [6].

Among these nanocarriers, mesoporous silica nanoparticles (MSNs) have garnered significant attention due to their unique physicochemical properties, including high drug loading capacity, tunable pore sizes, excellent stability, and ease of surface modification [7]. These characteristics make MSNs stand out compared to other nanocarriers, such as liposomes, polymeric micelles, and metallic nanoparticles. For instance, while liposomes and polymeric micelles are known for their biocompatibility, they often suffer from limited drug loading efficiency and premature drug release due to their inherent instability in vivo. Metallic nanoparticles, on the other hand, offer robustness and multifunctionality but can exhibit significant cytotoxicity and challenges in biocompatibility. In contrast, MSNs provide a balance of high stability, low toxicity, and the ability to carry a wide range of therapeutic molecules, making them an attractive platform for targeted drug delivery, particularly in the context of cancer treatment [8].

Cancer remains a leading cause of mortality worldwide, and conventional treatment methods often suffer from limitations, such as non‐specificity, systemic toxicity, and drug resistance. Therefore, the development of precise and effective drug delivery systems is imperative. MSNs offer a versatile solution by serving as multifunctional carriers that can be engineered to respond to specific stimuli within the tumor microenvironment [9]. These stimuli‐responsive MSNs enable the controlled and targeted release of therapeutic agents, thereby enhancing the therapeutic index and reducing adverse effects on healthy tissues.

Recent dual‐input logic‐gated nanoplatforms have further demonstrated that the requirement for simultaneous tumor‐associated signals, such as acidic pH and hypoxia, can improve tumor specificity and reduce systemic toxicity, supporting the rationale for the multi‐stimuli‐responsive MSN design [10].

This review aims to provide a comprehensive overview of the strategies and prospects for the design and application of stimuli‐responsive multifunctional MSNs in cancer therapy. We will explore the physicochemical properties of MSNs, surface functionalization strategies, and various stimuli‐responsive mechanisms that enable precise drug delivery. This review also discusses the challenges associated with the clinical translation of MSNs and highlights future research directions in this rapidly evolving field. By harnessing the unique capabilities of stimuli‐responsive multifunctional MSNs, we can pave the way for more effective and targeted cancer treatment, ultimately improving patient outcomes and advancing the field of nanomedicine.

## 2 PHYSICOCHEMICAL PARAMETERS THAT AFFECT MSN PERFORMANCE

The morphology, surface characteristics, and pore size of MSNs play critical roles in their properties [11]. Therefore, by identifying how the shape, size, surface functionalities, and pores influence biological processes, researchers can redesign MSNs to maximize their efficacy in specific biomedical applications.

### 2.1 Shape and morphology

Mesoporous silica nanomaterials have different shapes. For instance, film‐, platelet‐, sphere‐, rod‐, rope‐, and fiber‐like shapes, hollow/rattle‐type, and multi‐shell‐type MSNs have attracted considerable attention due to their unique spatial architectures and high potential in biomedical applications [12, 13]. The particle shape plays a critical role in realizing many MSN applications, especially cancer therapy [13]. Several research groups have examined the impact of the shape of MSNs on drug adsorption and release. Rod‐shaped MSNs have shown shape‐dependent advantages in drug delivery and antitumor applications, while mesoporous silica nanorods have also been developed as multifunctional cell‐imaging probes [14, 15]. Hao et al. synthesized film‐, platelet‐, sphere‐, and rod‐shaped MSNs by adjusting the ratio of the CTAB and TBAI surfactant templates. Their results showed shape‐dependent release behavior, with film‐shaped MSNs exhibiting the fastest release because of their short vertically oriented pore channels [12]. The shape of MSNs not only influences drug loading and release but also affects cytotoxicity. Huang et al. found that long rod‐shaped MSNs exhibit higher cytotoxicity than short rods and spheres, owing to their increased surface area and curvature, which enhance the interaction with cell membranes. This shape‐dependent cytotoxicity is linked to the disruption of the cytoskeleton by long rod‐shaped MSNs, leading to higher rates of apoptosis in cancer cells [16]. Controlling the shape of MSNs involves adjusting the concentrations of key reagents, such as ammonia, TEOS, and CTAB [17]. Zhang et al. demonstrated that different shapes, such as rods and spheres, can be synthesized by varying the introduction of small molecules, for example, n‐octanol, resulting in rod‐shaped and helical‐structured MSNs [18].

In addition, hollow MSNs (HMSNs) are a particularly advantageous subtype due to their large hollow cavities, low density, and tunable pore sizes, which provide excellent drug loading capacity and biocompatibility [19]. Yolk‐shell MSNs contain an encapsulated core within a hollow mesoporous silica shell, allowing the core and shell to perform separate functions. One‐pot and core–shell fabrication strategies have been developed to prepare these structures [20, 21].

### 2.2 Particle size

Controlling the particle size of MSNs is a critical issue because this parameter can significantly affect the efficacy of endocytosis, sensing, drug loading and release, cellular uptake, cytotoxicity, and the enhanced permeability and retention (EPR) effect [22]. Several studies have reported tuning particle size by controlling synthetic parameters such as temperature, reaction time, pH, and the addition of functional organosilanes or suitable additive agents such as amines, inorganic bases, inorganic salts, and alcohols. These agents lead to alterations in the condensation and hydrolysis of silica precursors and accelerate the reaction kinetics, resulting in smaller particle sizes [23]. To control the particle size of MSNs, several studies have utilized additional agents or unique treatments. Okubo et al. prepared colloidal MSNs with a particle size of 70–110 nm by adding ethylene glycol [24]. Qiao et al. synthesized MSNs with controlled particle sizes from 25 to 200 nm via adding alcohols and inorganic bases [25]. Tang et al. prepared drug–silica nanoconjugates with diameters of 20, 50, and 200 nm and found that the 20 nm particles showed greater cellular internalization, tumor accumulation, and tumor penetration than the 50 and 200 nm analogues [26].

### 2.3 Pore size and cargo loading

Pore size is crucial in biotechnology since it determines the ability to adsorb and release biomolecules effectively. By adjusting the pore architecture and surface chemistry of MSNs, a wide variety of drug molecules, including macromolecules, can be effectively loaded and released [27]. Pore size control is achieved by modifying the concentration or chemical structure of the templates and co‐templates, as well as by varying the dispersed phase parameters, such as temperature, pH, or ionic strength [28]. For example, an excess of the silica source can result in a disordered mesostructure, whereas insufficient silica may fail to form the desired mesoporous structure [29]. The concentration and type of surfactant are also crucial, as low surfactant concentrations may prevent micelle formation, leading to an inadequate pore structure, whereas excessively high concentrations can cause disorder [30]. Pore size can be further fine‐tuned during synthesis or through post‐synthesis hydrothermal treatment. Selecting surfactants with varying hydrophobic chain lengths or using swelling agents, such as mesitylene, allows for the formation of specific pore sizes [31]. For instance, doxorubicin (DOX)‐encapsulated MSNs with a pore size of 5.4 nm demonstrated superior cellular uptake and tumor inhibition compared to other sizes, highlighting the importance of optimizing pore dimensions for anticancer efficacy [32]. Additionally, researchers have developed MSNs with extra‐large pores (approximately 25 nm) to accommodate high molecular weight protein antigens [33], and even ultra‐large pores (about 47.5 nm) for improved adsorption of antibodies and large proteins, for instance, immunoglobulin G (IgG) and bovine serum albumin (BSA) [34].

### 2.4 Surface functionalization and molecular gatekeeping

The surface properties of MSNs can be tailored to enhance bioavailability, enable targeted delivery, and control the release of therapeutic molecules by introducing various functional groups. The robust honeycomb‐like framework of MSNs, with its large active surface area, allows for the attachment of diverse functional groups, facilitating improved biological interactions [35].

**FIGURE 1:**
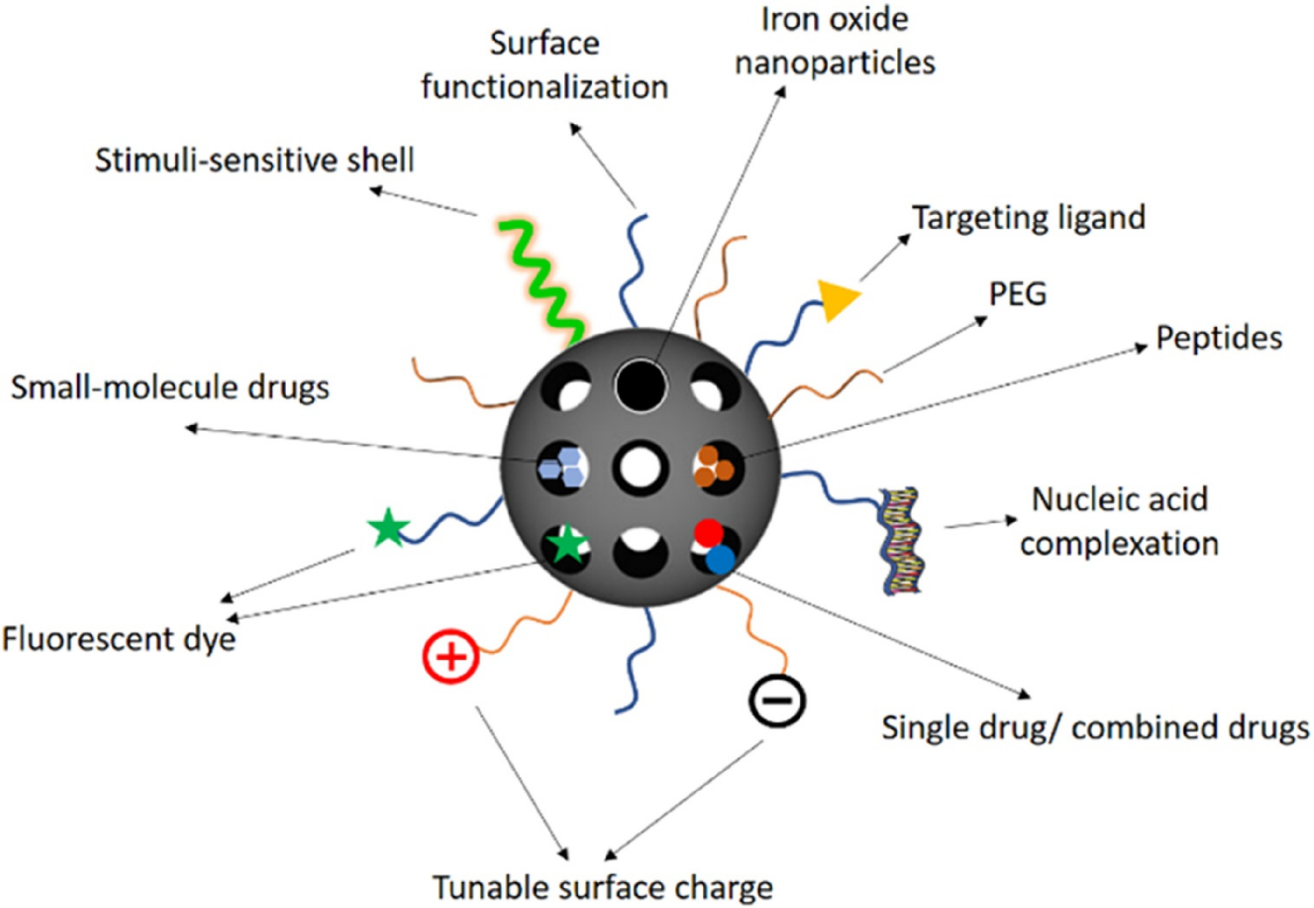
Illustration of various ways for surface functionalization and cargo loading in MSNs.

Common functionalization methods include the introduction of amine, thiol, carboxyl, and methyl groups. Amine groups can enhance the adsorption of drugs with acidic properties and can be introduced through post‐synthetic grafting or co‐condensation methods [36]. Thiol groups can be conjugated to the silica surface for applications such as drug anchoring, while carboxyl groups can increase the adsorption of basic drugs and serve as sites for further bioconjugation, such as coupling with fluorescent dyes [37]. Methyl group functionalization imparts hydrophobic characteristics to MSNs, influencing their interactions with biological environments [38]. However, studies have shown that amine‐functionalized MSNs generally exhibit higher cellular uptake due to favorable electrostatic interactions with negatively charged cancer cell membranes. Surface modification with dendritic polymers can promote endosomal escape and facilitate cytosolic cargo delivery [39]. Additionally, the bioconjugation of ligands such as antibodies [40], peptides [41], aptamers [42], and small molecules [43] can significantly enhance the specificity of MSNs for targeting cancer cells (Figure 1), improving their retention in tumors and facilitating endocytic uptake by target cells.

## 3 TUMOR‐TARGETING STRATEGIES OF MSNS

Targeted drug delivery technology has become an essential tool in anticancer therapy research to reduce side effects [44]. It mainly focuses on delivering cargo to the site of interest while decreasing off‐target effects; therefore, selective targeting increases efficacy and reduces undesired side effects [45]. Nanoparticles (NPs) face several hurdles and transport barriers on their way to the tumor site; for instance, rapid clearance via the reticuloendothelial system (RES), nonspecific uptake of healthy cells, and aggregation during circulation [46]. Tumor site‐directed therapy facilitates the reduction of both toxic side effects and drug doses and enhances treatment efficacy. Generally, targeting strategies are divided into passive targeting and active targeting.

### 3.1 Passive targeting

The passive targeting of MSNs is the mechanism by which the cargo accumulates in tumor tissue by exploiting the abnormalities of tumor vasculatures, such as lack of lymphatic drainage, angiogenesis, and aberrant vascular architecture, which lead to the EPR effect; in turn, MSNs passively reach tumor tissues [47]. The accumulation efficiency relies on the physicochemical properties of MSNs, such as shape, surface charge, and size [48]. Even NPs that can pass the vasculature and leak into the tumor are hindered by the interstitial tumor matrix, which creates a barrier to their deep penetration into tumor tissue. Decreasing the particle size of the nanosystem can significantly reduce diffusional hindrance, thereby enhancing its penetration into the interstitial matrix [49]. When injected intravenously, traditional NPs can be quickly cleared from the circulation (minutes) via the mononuclear phagocyte system (MPS) and localized in the liver, bone marrow, and spleen [50]. Surface modifications of MSNs with polyethylene glycol (PEG) can extend the circulation time of the NPs in the blood while reducing both the MPS recognition and the clearance rate of MSNs [51].

### 3.2 Active targeting

Active targeting strategies for MSNs are crucial for enhancing the delivery of therapeutic agents to tumor tissues, particularly in cases where the EPR effect is insufficient, such as pancreatic cancer. These strategies rely on specific interactions between ligands on the nanoparticle surface and overexpressed receptors on cancer cells, thereby improving the precision and efficacy of drug delivery [52].

Several common active targeting strategies involve the use of various ligands that bind to specific receptors on tumor cells. Aptamers, for instance, are single‐stranded DNA or RNA molecules that can fold into unique three‐dimensional shapes, allowing them to bind selectively to target molecules, that is, ATP or cell surface proteins [53]. Peptides, such as RGD, and proteins, including antibodies and transferrin, are also widely used to enhance the targeting specificity of MSNs [54]. Additionally, small molecules like folates and anisamide have been employed for their ability to target overexpressed receptors in certain cancer types [55]. Recent prostate‐specific membrane antigen (PSMA)‐targeted near‐infrared II (NIR‐II) molecular probes further demonstrate that small‐molecule ligand recognition can be integrated with high‐contrast optical imaging for tumor detection and image‐guided surgery, supporting the broader development of imaging‐capable active‐targeted MSN theranostic platforms [56].

These strategies significantly improve the accumulation of nanoparticles in tumor cells, thereby enhancing therapeutic efficiency while minimizing side effects on healthy tissues [57]. Beyond ligand‐receptor interactions, magnetic‐field‐directed targeting represents another powerful strategy. Researchers can guide nanoparticles to the tumor site using an external magnetic field by incorporating ferromagnetic nanoparticles into MSNs. This method is particularly effective for targeting tumors located near the body surface and offers the added benefit of noninvasive control over the drug delivery process [58]. Cell‐mediated targeting offers a novel strategy by utilizing the body's own circulatory cells to transport MSNs to the tumor site. This approach can exploit the long circulation lifespan of red blood cells (RBCs) and the natural homing abilities of other circulatory cells to improve MSN delivery efficiency. By hitchhiking on appropriate circulatory cells, MSNs can avoid rapid clearance and increase their chances of reaching diseased tissues [59].

While these active targeting strategies have shown significant promise, they often face challenges, such as insufficient targeting specificity and the need for better control over drug release. Stimuli‐responsive modifications to MSNs can overcome these challenges. By incorporating stimuli‐responsive elements that can react to specific conditions within the tumor microenvironment, such as pH, temperature, or enzyme activity, these modified MSNs can achieve more precise and controlled drug release. This approach not only enhances the targeting accuracy but also reduces off‐target effects, making stimuli‐responsive MSNs a critical focus of ongoing research and the subject of the next chapter.

## 4 STIMULI‐RESPONSIVE STRATEGIES

Many MSN designs have been evolved to undergo chemical structure change, biochemical, or physical property variations in response to environmental stimuli such as enzymes, light, pH, temperature, and ionic strength to obtain tumor‐specificity and highly controllable cargo release [60]. This approach decreases the drug interactions with normal tissues during its circulation in the body [61]. Strategies utilized for modification of the surface of MSNs with stimuli‐responsive moieties for preventing the premature release of cargo and selectively targeting the tumor site based on excluding the cargos within the tubular pores, which can be achieved by coating the MSN surface [62], pore‐blocking with molecular or particle gates [63], or cargo coupling to the inner wall of the pore [64]. These approaches depend on two kinds of responses: the conformational change or destruction of the pore‐blocking agent, or the utilization of labile/cleavable bonds that are broken upon stimulus exposure [65] by the presence of specific intrinsic cues of the inherent characteristic in the cancer microenvironment, or by application of certain extrinsic stimuli to the target site from outside the body [66].

### 4.1 Intrinsic stimuli‐responsive MSNs

**FIGURE 2:**
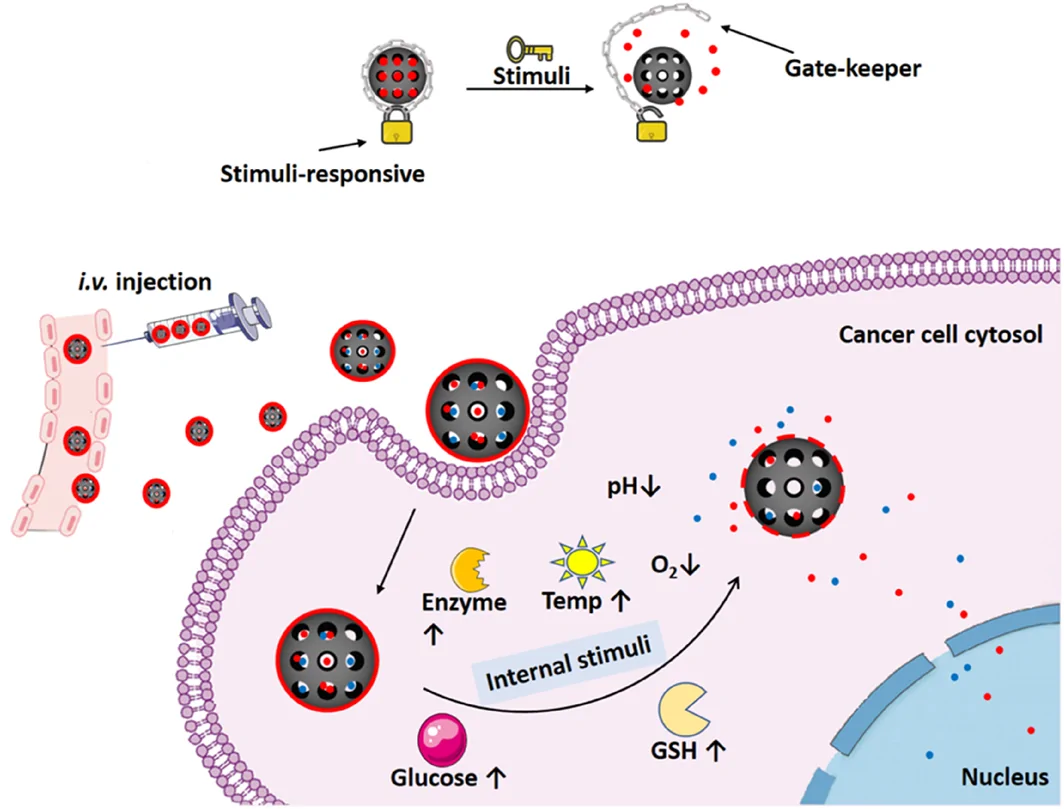
Schematic illustration of abnormal internal conditions in tumor cells, which can be exploited as internal stimuli to trigger cargo release in tumors.

The internal stimuli of chemical and biochemical origins comprise pH‐, redox‐, enzyme‐, ionic strength‐, temperature‐responsive drug delivery nanosystems, and others. These stimuli‐sensitive NPs trigger cargo release in response to the abnormal conditions in the microenvironment of tumor tissues, which are significantly different from those in healthy tissues (Figure 2); for instance, overexpression of specific enzymes, lower pH, or higher temperature compared with healthy tissues [67]. Examples of internal stimuli‐responsive MSNs are shown in Table 1.

**TABLE 1:** Representative intrinsic stimuli‐responsive MSN systems and their quantitative release/therapeutic outcomes.

Stimulus	Formulation	Cargo	Gatekeeper	Triggering condition	Release behavior	Cancer model/study type	Therapeutic/imaging outcome	Ref
pH	UA@MSNs‐CS‐LA SO@MSNs‐CS‐LA	UA or SO	Chitosan‐lactobionic acid coating; acid‐labile amide bond (CS‐LA)	pH 5.5 versus pH 7.4	UA@MSN‐CS‐LA: 26.5% UA release at pH 7.4 vs. 65.3% at pH 5.5 after 96 h. SO@MSN‐CS‐LA: 24.9% SO release at pH 7.4 vs. 65.0% at pH 5.5 after 96 h. Uncoated MSN‐COOH released ∼79%–81% under both pH conditions, showing weaker gatekeeping	SMMC‐7721, HepG2, HeLa; H22 tumor and lung metastasis models	IC50 in SMMC‐7721: UA + SO 10.05 μM versus USMNs‐CL 5.83 μM; apoptosis: UA 18.83%, SO 22.73%, UA + SO 54.45%, USMNs‐CL 70.49%; adhesion reduced to 31.3% and migration to 32.18%; higher tumor accumulation and reduced lung metastasis	[68]
pH	TOP@MSNs‐G‐FA	Topotecan	Gelatin coating	pH 5.3 versus pH 7.4	MSN‐FA(TOP): ∼3.1% TOP release at pH 7.4 vs. ∼8.7% at pH 5.3 after 48 h	LNCaP cancer cells versus MC3T3‐E1 normal‐like cells	Blank MSNgel/MSN‐FA showed >99% viability; FA‐modified TOP‐loaded MSNs showed selective uptake and cytotoxicity in folate receptor‐positive LNCaP cells compared with MC33‐E1 normal‐like cells.	[69]
pH	DOX‐BA@MSNs‐PEG	DOX	DOX‐BA	Weakly acidic pH < 6.8 versus pH 7.4	M‐CHO‐DOX@DOX‐PEG: 10.6% DOX release at pH 7.4, 55.3% at pH 6.8%, and 86.4% at pH 5.4. Non‐PEG M‐CHO‐DOX@DOX showed similar release: 58.1% at pH 6.8% and 85.4% at pH 5.4.	HeLa cells; HeLa xenograft model	pH 6.8 increased cellular uptake; M‐CHO‐DOX@DOX‐PEG showed a 2.3‐fold increase in intracellular DOX fluorescence at pH 6.8 compared with pH 7.4, lower toxicity at pH 7.4, stronger cytotoxicity at pH 6.8, and superior in vivo antitumor efficacy without obvious body‐weight loss.	[70]
Enzyme	RhB@PLDA‐MSNs DOX@PLDA‐MSNs	RhB or DOX	Polylysine‐dopamine coating (PLDA)	Pepsin, pH 1.7; 5 U or 40 U	∼10% DOX release at pH 7.4 or pH 1.7 without pepsin over 114 h; ∼30% release with 5 U pepsin; ∼56% release with 40 U pepsin at pH 1.7	HeLa cells; Marc‐145 cells for biocompatibility	PLDA‐MSNs showed ∼96% viability at 50 μg/mL in Marc‐145 cells; DOX@PLDA‐MSNs produced dose‐dependent HeLa inhibition, although weaker than free DOX because of sustained release	[71]
Enzyme	Ru‐tLys‐S1/CPT‐tLys‐S1; Ru‐rLys‐S1/CPT‐rLys‐S1	CPT or [Ru(bipy)_3_]^2+^	ε‐poly‐L‐Lysine	Protease from S. griseus, 0.12 mg/mL	No significant dye release without protease. With protease, Ru‐tLys‐S1 released 69% and Ru‐rLys‐S1 released 91% of maximum dye release after 6 h	HeLa and MCF‐7 cells	Enzyme‐gated intracellular CPT release induced cell death in HeLa and MCF‐7 cells; cytotoxicity was evaluated by live‐cell imaging/flow‐based assays rather than simple release percentage	[72]
Redox/enzyme	Fe_3_O_4_/DOX@R‐S‐S‐R_1_‐MSNs	DOX	R‐S‐S‐R_1_	GSH/glutathione‐reductase‐related reducing environment	Without GSH, DOX release was below 25% after 24 h. With 10 mM GSH, DOX release reached 94.89%, 69.06%, and 42.76% after 24 h for R‐S‐S‐R1‐1, R‐S‐S‐R1‐2, and R‐S‐S‐R1‐3, respectively. At 4 h, the release was 45.16%, 36.50%, and 10.90%, respectively	HeLa cells; HUVEC control	HeLa uptake was observed; HUVEC cytotoxicity was approximately 28.4% at 50 μg/mL, indicating limited toxicity to normal endothelial cells	[73]
Redox	BHQ‐MSNs‐SS‐PpIX&RGD	PpIX	KGK(peptide)‐PEG	10 mM GSH, pH 7.4, 37°C	The release was not reported as a conventional percentage. Instead, 10 mM GSH caused a ∼3‐fold increase in PpIX fluorescence after 96 h, indicating GSH‐triggered disulfide cleavage and fluorescence recovery	HeLa cells	RGD‐mediated uptake and fluorescence recovery enabled tumor‐specific imaging and enhanced PDT	[74]
Redox	DOX@MSNs‐S‐S‐Tf	DOX	Tf	10 mM GSH; 2 mM GSH; pH 7.4, 6.5, and 5.0	With 10 mM GSH, DOX release was >50% within 3.5 h and ∼63% within 24 h. Without GSH, the initial leakage was ∼12%, reaching ∼22.1% over 24 h	Huh7 cells	Blank MSNs‐S‐S‐Tf showed >90% viability at 1–500 μg/mL, and the DOX‐loaded system showed dose‐dependent cytotoxicity and Tf‐mediated targeting	[75]
Temperature	PNIPAAm/Au@MSNs	Au nanoparticles (catalyst)	PNIPAAm	30°C versus 50°C, relative to LCST	Temperature‐regulated catalytic activity; turnover frequency (TOF) was 14.8 h^−1^ at 30°C versus 2.4 h^−1^ at 50°C	Non‐cancer catalytic model	Temperature‐responsive nanoreactor model; no therapeutic efficacy evaluated	[76]
Temperature	Safranin@MSNs‐P	Safranin O dye	Peptide	Temperature‐induced peptide secondary‐structure change	Release was negligible from 4°C to ∼40°C; heating to 90°C caused complete Safranin O release. Reloaded S1‐P showed <4% release at 4°C over 12 h	Model‐dye release study	No cancer efficacy evaluated	[77]
ROS	DOX@MSNs‐Gd‐DOTA	DOX	Gd‐DOTA	660 nm laser, 0.5 W/cm^2^, 10 min	13% DOX release without irradiation versus 76% with laser‐generated ROS over 3 days; ungated DOX‐MSN released 29% without irradiation	Cancer cells; tumor‐bearing mice	IC50: 73.1 μg/mL with laser vs. 164.3 μg/mL without laser; MR imaging confirmed tumor accumulation and tumor growth was significantly suppressed	[78]
Hypoxia	DOX@MSNs‐CD‐HR	DOX	NI‐CD	Hypoxia versus normoxia	DOX‐CD‐HR‐MSN released 10% DOX under normoxia vs. 84% under hypoxia after 3 days	SCC‐7 cells	IC50: 33.7 μg/mL under hypoxia versus 60.3 μg/mL under normoxia; confocal imaging confirmed intracellular DOX release under hypoxia	[79]
Hypoxia	Ce6@MSNs‐CpG/ODN‐PEG	Ce6 + CpG	CpG/GC	Tumor hypoxia below 2% O_2_; PDT‐induced oxygen consumption	CAGE released ∼70% of the loaded CpG under hypoxic conditions, whereas <10% was released under normoxia. Hypoxia‐nonresponsive CUGE showed no significant normoxia/hypoxia difference	B16.Mo5/B16.F1 melanoma model; dendritic‐cell immune assays	CAGE + laser increased CD11c + MHCII + DC recruitment, whereas CAGE/CpG increased CD80+CD86+ DC activation. CAGE/CpG + laser produced long‐lasting tumor‐growth inhibition and 100% survival over 28 days	[80]
Hypoxia	Ce6@MSN/pDAB/F68; DOX@MSN/pdab/F68	Ce6 or DOX	pDAB/F68	Hypoxia/sodium dithionite; 660 nm laser for Ce6 PDT	For FRET cargo, release was monitored by decreasing the FRET ratio rather than direct release. For DOX@MSN/pDAB/F68 under mimicked hypoxia: ∼20% release at 0 mM Na_2_S_2_O_4_, ∼58% at 5 mM, and ∼100% at 10 mM after 24 h	MCF‐7 cells; 4T1 tumor‐bearing mice	Ce6 IC50: Free Ce6 3.5 ± 0.4 μg/mL under normoxia and 5.2 ± 0.1 μg/mL under hypoxia; Ce6@MSN/pdab/F68 6.4 ± 0.8 μg/mL under normoxia and 8.2 ± 1.1 μg/mL under hypoxia. DOX@MSN/pDAB/F68 IC50: 3.7 ± 0.2 μg/mL under hypoxia versus 5.3 ± 0.5 μg/mL under normoxia	[81]
Glucose	MSNs‐GOx/PLL/HA	GOx and PTX	PLL gate + HA targeting shell	Glucose‐mediated GOx reaction producing gluconic acid/H_2_O_2_; pH 5.0, 6.5, and 7.4	PTX release was <15% at pH 7.4, ∼42% at pH 6.5, and ∼78% at pH 5.0 after 24 h	HepG2 and MCF‐7; xenograft tumor model	GOx increased intracellular H_2_O_2_ and apoptosis in HepG2 cells in a glucose‐dependent manner; saline‐treated tumors increased ∼20‐fold in volume, whereas MSNs‐GOx/PLL/HA markedly inhibited tumor growth	[82]
Glucose	Fe@MSNs‐PBA‐dextran‐FA/FA‐MMS‐dextran	Tolbutamide or CPT	Dextran gate bound to phenylboronic acid; FA targeting for cancer	Glucose competitive binding to PBA	CPT: ∼27% release without glucose, ∼36% at 4 mg/mL glucose, ∼58% at 10 mg/mL, and ∼82% at 50 mg/mL after ∼500 min of incubation. Tolbutamide: ∼12% without glucose, ∼30% at 4 mg/mL, ∼58%–60% at 10 mg/mL, and ∼78%–80% at 50 mg/mL after ∼720 min	HeLa cancer cells; RIN‐5F β‐cells	CPT‐loaded FA‐MMS‐dextran reduced HeLa viability to ∼40% in glucose‐containing medium; viability was >70% in glucose‐free medium; and blank carriers maintained >75% viability	[83]
ATP	TDPA‐Zn^2+^‐UCNP@MSNs	DOX or CPT	Poly(Asp‐Lys)‐*b*‐Asp	ATP sensing: 50 μM–1 mM; model‐release assays: 1–10 mM ATP; 5 mM ATP for DOX/CPT monitoring	With 5 mM ATP, DOX release reached ∼85% and CPT release ∼64% after 24 h. Model fluorescein release also showed ATP dependence: ∼57% with 1 mM ATP, ∼80% with 5 mM ATP, and ∼91% with 10 mM ATP after 240 min, with negligible release without ATP	Release‐monitoring study	Therapeutic efficacy not evaluated; ATP‐triggered DOX/CPT release and luminescence resonance energy transfer (LRET)‐based real‐time monitoring	[84]
ATP	Aptamer‐MSNs	[Ru(bipy)_3_]^2+^ dye	ATP aptamer + arm ssDNA gate	20 mM ATP for 7 h	Negligible leakage without ATP; 83.2% release with 20 mM ATP after 7 h; 21.2% release from control DNA‐MSN	ATP‐responsive model‐dye study	No cancer efficacy evaluated	[85]
Ionic strength	IBU@MSNs IBU@MSNs‐PAH/PSS	Ibuprofen	PAH/PSS polyelectrolyte multilayer	NaCl 10 versus 0.5 mM	∼80% release at 10 mM NaCl over 48 h versus ∼13% leakage at 0.5 mM	Non‐cancer ibuprofen model	No anticancer efficacy evaluated	[86]
Urea	DOX@MSNs/GA‐urease nanobots	DOX	Glutaraldehyde (GA)‐urease	Urea 10–100 mM; saturation around 50 mM	DOX release increased with urea and saturated around 50 mM urea. ∼6% release without urea *vs* ∼24%–25% release at 50–100 mM urea after 24 h, approximately a 4‐fold increase	HeLa cells	DOX‐loaded active nanobots achieved comparable HeLa killing at 0.05 mg/mL to passive nanobots at 0.5 mg/mL, representing about a 10‐fold lower carrier dose; urea increased intracellular DOX fluorescence and cytotoxicity	[87]

Abbreviations: ATP, adenosine triphosphate; BHQ, black‐hole quencher; Ce6, chlorin e6; CPT, camptothecin; CS‐LA, chitosan–lactobionic acid; DOX, doxorubicin; FA, folic acid; FRET, Förster resonance energy transfer; GA, glutaraldehyde; GC, glycol chitosan; Gd‐DOTA, gadolinium–DOTA complex; GOx, glucose oxidase; GSH, glutathione; HA, hyaluronic acid; HUVEC, human umbilical vein endothelial cells; IBU, ibuprofen; LCST, lower critical solution temperature; LRET, luminescence resonance energy transfer; NI‐CD, nitroimidazole–cyclodextrin; PAH, poly(allylamine hydrochloride); PBA, phenylboronic acid; PDT, photodynamic therapy; PEG, polyethylene glycol; PLDA, polylysine–dopamine; PLL, poly(L‐lysine); PNIPAAm, poly(N‐isopropylacrylamide); PpIX, protoporphyrin IX; PSS, poly(styrene sulfonate); PTX, paclitaxel; RGD, Arg‐Gly‐Asp; RhB, rhodamine B; ROS, reactive oxygen species; SO, sorafenib; TDPA‐Zn^2+^, zinc–dipicolylamine analogue; Tf, transferrin; TOF, turnover frequency; TOP, topotecan; UA, ursolic acid; UCNP, upconversion nanoparticle.

*pH‐responsive MSNs*: pH‐responsive nanosystems have garnered significant attention in cancer therapy due to the acidic microenvironment of tumor tissues, which contrasts with the neutral pH of healthy tissues. The extracellular pH of solid tumors is typically lower than that of the surrounding normal tissues, often below 6.5, providing an opportunity to exploit this difference for targeted drug delivery. By functionalizing the surface of MSNs with pH‐sensitive components that act as gatekeepers, the release of antitumor drugs can be triggered specifically under acidic conditions of the tumor microenvironment [88].

Lee et al. developed a novel pH‐responsive system using poly(L‐lysine) (PLL)‐gated MSNs for the delivery of DOX. The PLL layer remained dense and impermeable under physiological pH, effectively preventing premature drug release. However, in the acidic environment of tumors, the PLL layer swelled and DOX release reached approximately 85% within 24 h, about 1.5‐fold higher than at physiological pH [89]. Zhao et al. advanced this concept by developing a pH‐responsive system for the co‐delivery of ursolic acid (UA) and sorafenib (SO). They functionalized MSNs with carboxyl groups and coated them with chitosan‐lactobionic acid (CS‐LA), forming an acid‐labile amide bond. The system showed minimal drug release under physiological conditions, but when exposed to the acidic tumor environment (pH 5.5), the release rates of UA and SO increased significantly, demonstrating substantial anticancer effects. In vitro studies revealed that the pH‐responsive system had higher cytotoxicity against cancer cells compared to non‐gated MSNs, highlighting its potential for enhanced cancer treatment [68]. Martinez‐Carmona et al. introduced an enhanced pH‐responsive system using gelatin‐coated MSNs for the delivery of topotecan (TOP). The gelatin coating acted as a pH‐sensitive gatekeeper, preventing premature drug release and allowing for selective targeting of tumor cells. The system released more TOP at pH 5.3 than at pH 7.4; after 48 h, the release was approximately 8.7% and 3.1%, respectively. This behavior was attributed to the pH‐sensitive gelatin coating [69]. A novel strategy by Zeng et al. employed a drug self‐gated MSN system using a pH‐sensitive dynamic benzoic‐imine covalent bond. This innovative approach used the drug itself, in this case, DOX, as both the cargo and the gatekeeper, eliminating the need for auxiliary capping agents. The system showed controlled and on‐demand release of DOX under weakly acidic conditions (pH < 6.8) typical of tumor cells, significantly enhancing antitumor efficacy while minimizing premature drug release [70]. Wang et al. further explored pH‐responsive MSNs by integrating glucose oxidase and copper ions into HMSNs for selective tumor starvation and chemodynamic therapy (CDT). The system exploited the acidic tumor microenvironment to promote the release of Cu ions, which reacted with H_2_O_2_ to produce highly cytotoxic hydroxyl radicals (•OH) through a Fenton‐like reaction. This approach led to significant tumor growth inhibition, showcasing the potential of pH‐responsive MSNs for effective cancer therapy [90].

In general, pH‐responsive MSNs are broadly useful because they exploit extracellular tumor acidity and stronger endo/lysosomal acidification after cellular uptake. The limitation of this approach is that tumor acidity is heterogeneous and often mild; therefore, the gate should respond within physiologically realistic pH ranges. These systems are most suitable for acidic tumors or carriers designed for endosomal release.

*Enzyme‐responsive MSNs*: Enzyme‐responsive MSNs exploit differences in enzyme expression or activity between tumors and normal tissues. Tumors often overexpress enzymes, such as matrix metalloproteinases (MMPs), hyaluronidase (HAase), cathepsins, urokinase, or other hydrolases, because of extracellular matrix remodeling, invasion, inflammation, and altered metabolism [91, 92]. These enzymes can degrade gatekeepers or cleave peptide/polymer linkers, thereby triggering selective cargo release in enzyme‐rich tumor regions. The main advantage of this strategy is its biochemical specificity under mild conditions; however, its performance depends on enzyme abundance, spatial distribution, substrate accessibility, and intertumoral heterogeneity.

**FIGURE 3:**
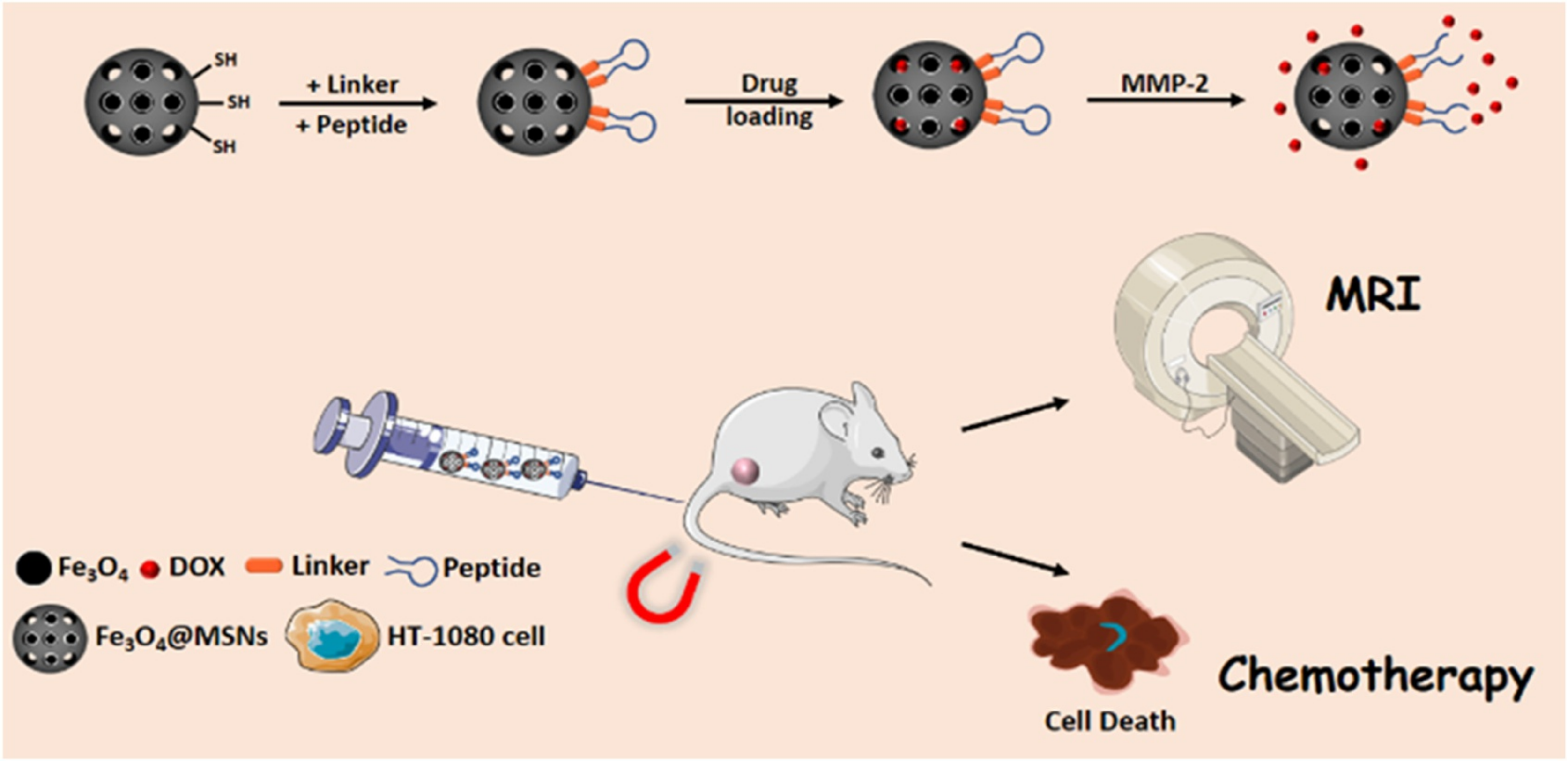
Schematic illustration of peptide‐Fe_3_O_4_@MSNs for enzyme‐responsive drug delivery and magnetic resonance imaging (MRI).

Hu et al. developed a pepsin‐responsive platform based on polylysine‐dopamine (PLDA)‐coated MSNs. The PLDA coating was formed through the self‐polymerization of lysine‐dopamine and served as an enzyme‐degradable gatekeeper. The resulting RhB/DOX@PLDA‐MSNs nanosystem encapsulates Rhodamine B (RhB) or DOX and releases these cargos after pepsin‐mediated degradation of the PLDA coating under acidic conditions. The system demonstrated that pepsin significantly induced DOX release, with approximately 30% and 56% of DOX released in the presence of 5 and 40 U of pepsin, respectively, within 114 h. In contrast, only 10% of DOX was released at pH 7.4 and 1.7 without pepsin, highlighting the stability of the peptide bonds under non‐enzyme conditions [71]. Mondragon et al. designed an MSN system in which ɛ‐poly‐L‐lysine was covalently attached to the MSN surface as a gatekeeper for [Ru(bipy)_3_]^2+^ or camptothecin (CPT)‐loaded MSNs. This polymer was attached using two different strategies: random attachment through nonspecific reactions of the polymer's amino groups with MSNs (Ru‐rLys‐S1) and specific attachment through the peptide's C‐terminal end (Ru‐tLys‐S1). When a protease enzyme was introduced, 69% and 91% of the dye was released from Ru‐tLys‐S1 and Ru‐rLys‐S1, respectively, due to the hydrolysis of amide bonds. To confirm the enzyme sensitivity, the enzyme was denatured by heating to 60°C for 1 h before introducing the nanosystems, and both nanosystems were incubated with other enzymes. In both cases, no dye release was detected, demonstrating the selective role of the protease enzyme in the gate‐opening mechanism [72]. Naz et al. developed HAase‐responsive MSNs by modifying them with triphenylphosphine (TPP) for mitochondrial targeting and further modifying them with hyaluronic acid (HA) to enhance active targeting of cancer cells via CD44 receptor‐mediated recognition. The HA also blocked the MSN pores to prevent premature drug release. In the absence of the HAase enzyme, less than 30% of DOX was released within 72 h. In contrast, 60% of the drug was released in the presence of HAase, approaching the release rate of non‐capped MSNs (70%). This feature reduces premature DOX release during blood circulation, minimizing side effects on healthy tissues. Confocal laser scanning microscopy revealed that most cells remained viable with MSNs alone, while DOX‐loaded HA‐modified MSNs resulted in only 34% cell viability in cancer cells (MGC‐803) after 48 h, confirming the system's good anticancer effect with lower cytotoxicity to healthy cells [93]. Chen et al. developed an HAase‐responsive theranostic MSN system integrating TPZ delivery, CD44 targeting, and multimodal imaging. HA functioned as both a targeting ligand and an enzyme‐degradable gatekeeper, with HAase triggering substantial release of TPZ and the photosensitizer while minimizing premature leakage. The platform combined PDT with hypoxia‐activated TPZ chemotherapy and achieved enhanced antitumor activity in vitro and in SCC‐7 tumor‐bearing mice, together with NIR fluorescence/MR imaging capability and low systemic toxicity [94]. Paredes et al. developed an enzyme‐responsive MSN‐based system using a GFLG peptide‐responsive linker. MSNs were tethered with an organotin‐based metallodrug and modified with folic acid (FA) to enhance accumulation in breast cancer cells, resulting in higher cytotoxicity. This system showed enhanced anticancer activity by inhibiting tumor growth and reducing hepatic and renal toxicity caused by off‐target effects with frequent administration [95]. Li et al. proposed a new design for magnetic drug delivery using multifunctional iron oxide nanoagents coated on MSNs (Fe_3_O_4_@MSNs). The surface of these MSNs was functionalized with the PLGVR peptide, an MMP‐2‐responsive substrate. This study demonstrated that the PLGVR‐Fe_3_O_4_@MSNs system allows monitoring of cellular drug release in vivo, as well as fluorescence imaging and magnetic resonance imaging (MRI) capabilities (Figure 3) [96].

Overall, enzyme‐responsive MSNs offer strong biochemical specificity because the release depends on enzymatic cleavage or degradation of the gatekeeper. However, enzyme expression varies among tumors and even within different tumor regions. They are most suitable when the target enzyme is validated and accessible in the tumor microenvironment or intracellular compartment.

*Redox‐responsive MSNs*: Redox‐responsive nanoparticles exploit the significant differences in redox potential between tumors and healthy tissues to achieve targeted on‐demand drug release at cancer sites. In cancer cells, the concentration of reducing agents, such as glutathione (GSH), is approximately three times higher than that in healthy cells, particularly in the cytosol, mitochondria, and nucleus, where it ranges from 2–20 mM. This is in stark contrast to the much lower concentrations found in the blood and extracellular matrix, which are approximately 100–1000 times lower (2–20 μM) [88, 91, 97].

For example, Yuan et al. developed a theranostic nanosystem using MSNs doped with black‐hole quenchers (black‐hole quencher) and loaded with the photosensitizer protoporphyrin IX (PpIX). To enhance its circulation half‐life, PpIX was PEGylated. The nanosystem also included the tumor‐targeting ligand RGD, which was conjugated to the PEGylated PpIX, enabling active targeting through the strong interaction with the αvβ3 integrin receptor, which is commonly overexpressed on tumor cells. Upon receptor‐mediated endocytosis, the system achieved fast recovery of PpIX fluorescence and enhanced photodynamic therapy (PDT) due to the GSH‐triggered transformation of the multifunctional BHQ‐doped MSNs. In experiments, GSH‐triggered PpIX fluorescence recovery was monitored in the presence of 10 mM GSH in PBS at pH 7.4 and 37°C over 15 min to 96 h. After 96 h, the fluorescence intensity increased threefold compared to that of the control (15 min), indicating efficient cleavage of disulfide bonds by the reducing action of GSH [74]. In another study, Chen et al. developed transferrin (Tf)‐modified DOX‐loaded MSNs, which showed significant redox sensitivity. Interestingly, Tf served as both a gatekeeper and a targeting agent without the need for additional reductant moieties. The redox‐responsive drug release was facilitated by Tf's conjugation with disulfide bonds. A rapid drug release was observed in the presence of 10 mM GSH, with 50% of the drug released within only 3.5 h and a total cumulative release of 63% within 24 h. In contrast, only 12% of DOX leaked in the absence of GSH, confirming the effective redox‐responsiveness of the disulfide bonds and the gatekeeping efficiency of Tf. The relatively low total drug release (∼63%) was attributed to the attractive interactions between the drug and NPs. This protein/MSNs‐based on‐demand drug delivery system demonstrated excellent biocompatibility across a broad concentration range, improved intracellular accumulation, and enhanced targeting capability in vitro, making it a promising candidate for future clinical applications [75]. Moreover, Xiao et al. achieved zero‐leakage drug delivery by designing a redox‐responsive MSN system that incorporated stearic acid's alkyl chain with a thiol group into DOX‐loaded MSNs. An RGD‐modified amphiphilic peptide was then conjugated as a gatekeeper. This gatekeeper effectively prevented any DOX release in the absence of a reducing agent. However, in the presence of dithiothreitol (DTT) or GSH, the peptide gate was cleared through disulfide bond cleavage, allowing controlled drug release [98].

In general, redox‐responsive MSNs are especially useful for intracellular release because reducing agents are much more abundant inside cells than in blood or extracellular fluids. Their main limitation is that redox gradients are not exclusive to cancer cells. These systems are best suited for carriers that enter tumor cells before releasing their cargo.

*ROS‐responsive MSNs*: Reactive oxygen species (ROS)‐responsive MSNs exploit the oxidative imbalance between tumor and normal tissues. Many tumor cells exhibit significantly higher ROS levels than healthy cells, with reported concentrations reaching up to approximately 100 μM, about 100‐fold higher than those in healthy cells. This biochemical difference provides a basis for tumor‐specific ROS‐triggered drug release. In MSN systems, ROS‐sensitive motifs are usually incorporated as gatekeepers, linkers, or degradable coatings that remain stable under physiological conditions but undergo cleavage or structural transformation under oxidative stress. Common ROS‐responsive chemistries include thioketals, thioethers, selenium/tellurium‐containing groups, boronic esters, peroxalate esters, amino acrylates, and polyproline‐based structures [99, 100, 101, 102, 103, 104]. Since ROS levels vary substantially across tumor types and treatment conditions, ROS‐responsive MSNs are most effective when combined with ROS‐generating modules, such as photosensitizers or chemodynamic components, rather than relying solely on basal tumor ROS. For example, Rao et al. developed ROS‐responsive MSNs (R‐MSNs) for MRI‐guided photodynamic chemotherapy. This system incorporated the gadolinium‐DOTA (Gd‐DOTA) complex as a ROS‐sensitive gatekeeper and PEG‐conjugated chlorin e6 (Ce6) as the ROS generator. The Gd‐DOTA complex, conjugated to the MSN surface via an amino acrylate linkage, is specifically cleaved by ^1^O_2_, enabling controlled drug release. Under laser irradiation, the R‐MSNs facilitated the release of 76% of DOX within 3 days, with only 13% released in the absence of ROS, demonstrating the effective gatekeeping function of the Gd‐DOTA complex. The IC50 of the ROS‐responsive DOX‐loaded MSNs with laser treatment was 73.1 μg/mL, significantly lower than the 164.3 μg/mL observed without laser irradiation, highlighting the efficacy of the ROS‐induced drug release [78]. In another study, Li et al. synthesized mPEG‐modified thioketal as a ROS‐responsive gatekeeper conjugated to MSNs, focusing on the antibacterial activity of the loaded cargo. Although this study targeted antibacterial applications, the ROS‐responsive environment closely mirrors that of the tumor microenvironment, suggesting the potential for adapting this nanosystem for anticancer drug delivery [105].

Collectively, ROS‐responsive MSNs are valuable when oxidative stress or treatment‐generated ROS can trigger release. Their advantage is that ROS can act as both a therapeutic mediator and a release signal. Because basal ROS levels vary among tumors, these systems are strongest when combined with PDT, CDT, or other ROS‐amplifying strategies.

**FIGURE 4:**
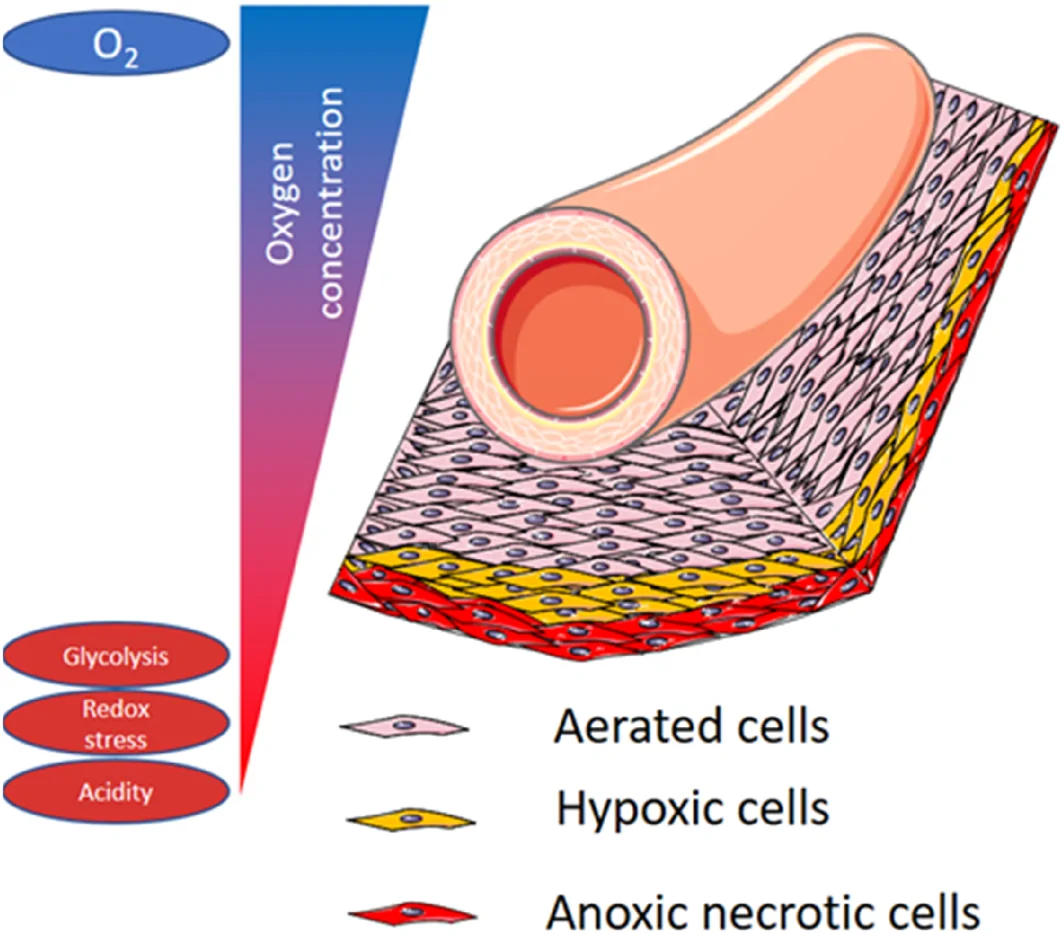
Illustration of the relationship between oxygen level and the distance of hypoxic tissues from the vein. A longer distance indicates more oxygen deficiency and leads to hypoxic and necrotic tissues.

*Hypoxia‐responsive MSNs*: Hypoxia‐responsive MSNs exploit the oxygenation differences between solid tumors and normal tissues. In many tumors, rapid proliferation and abnormal vasculature create oxygen gradients, whereas oxygen partial pressure can decrease to approximately 0–2.5 mm Hg in hypoxic tumor regions (Figure 4), compared with approximately 30–50 mm Hg in healthy tissues. This difference supports the use of hypoxia‐sensitive groups that remain relatively stable under normoxic conditions but undergo reduction or structural transformation in low‐oxygen tumor regions. Typical hypoxia‐responsive motifs include nitroimidazole, azobenzene, quinone, aromatic N‐oxide, aliphatic N‐oxide, and selected transition‐metal complexes. These systems are especially relevant for poorly vascularized tumors and PDT‐combination platforms, where oxygen consumption can further intensify local hypoxia. However, hypoxic regions are often distant from functional blood vessels, which may limit nanoparticle penetration. Therefore, hypoxia‐responsive MSNs are most effective when paired with penetration‐enhancing, active‐targeting, or imaging‐guided strategies [106, 107, 108, 109].

For example, Khatoon et al. developed a hypoxia‐responsive drug delivery system using MSNs loaded with DOX and a nitroimidazole‐cyclodextrin (NI‐CD) complex as a hypoxia‐sensitive gatekeeper. This system demonstrated effective drug release in the hypoxic tumor microenvironment through the bioreduction of the nitroimidazole stalk, confirming the potential of MSNs in hypoxia‐responsive drug delivery [79]. Im et al. designed a hypoxia‐responsive photosensitizer adjuvant co‐delivery system with Ce6‐doped azobenzene‐glycol chitosan (GC)‐PEG MSNs (CAGE). The system effectively utilized the hypoxic conditions to cleave an azo linker, leading to the release of CpG/GC complexes and demonstrating significant tumor inhibition through PDT‐assisted immunotherapy [80]. Yan et al. developed smart gated MSNs responsive to low oxygen levels by utilizing an azobenzene polymer as the gatekeeper. Under hypoxic conditions, the azo bonds broke, releasing the loaded Ce6 and demonstrating controlled drug release in the tumor environment [81, 110]. Recently, Jang et al. reported MSNs modified with a hypoxia‐responsive moiety, 4‐(phenylazo) benzoic acid, and β‐cyclodextrin as a gatekeeper. In hypoxic environments, the system released 80% of its DOX payload, showing significantly higher cytotoxicity to cancer cells compared to normoxic conditions [111].

In this context, hypoxia‐responsive MSNs are useful for poorly oxygenated tumors and PDT‐related systems, where oxygen consumption can further exacerbate hypoxia. Their limitation is the poor access of nanoparticles to severely hypoxic regions because these areas are often far from blood vessels. Therefore, they are best combined with penetration‐enhancing, targeting, and imaging‐guided strategies.

*Glucose‐responsive MSNs*: Glucose is a key stimulus that regulates drug release by responding to changes in its concentration, resulting in structural alterations in the delivery systems. Glucose oxidase (GOx) is widely used as a glucose‐responsive biocatalyst in glucose‐responsive nanosystems. GOx is a homodimeric enzyme consisting of two identical 80 kDa subunits, each containing a flavin‐adenine dinucleotide complex. When glucose is converted to gluconic acid via the GOx‐catalyzed reaction, the pH in the microenvironment of the nanoparticles decreases, leading to the swelling or shrinking of the GOx‐incorporated nanosystems or acidic degradation of the GOx‐containing polymer matrices, thereby triggering the release of the encapsulated drug [112].

In addition to GOx, glucose‐sensitive nanosystems utilizing concanavalin A (Con A) and phenylboronic acid (PBA) have been developed for self‐regulated drug delivery [113]. For example, Du et al. designed multifunctional MSNs decorated with poly‐L‐lysine (PLL) and HA for the co‐delivery of GOx and paclitaxel (PTX) for cancer treatment. The study demonstrated that GOx not only triggers intracellular glucose consumption to disrupt the energy supply but also increases endogenous H_2_O_2_ levels, thereby enhancing the therapeutic effects. PTX release was below 15% at pH 7.4, approximately 42% at pH 6.5, and approximately 78% at pH 5.0 after 24 h. GOx additionally consumed glucose and generated H_2_O_2_, supporting the combined starvation and chemotherapy effects. Tumors treated with saline showed a 20‐fold increase in volume compared to the first day of treatment, while the glucose‐responsive MSNs achieved significant tumor growth inhibition owing to the synergistic effects of H_2_O_2_ generation and chemotherapy [82]. Similarly, Sinha et al. developed a glucose‐responsive system by conjugating dextran with boronic acid‐modified MSNs. In this system, glucose binds to phenylboronic acid, causing the detachment of dextran chains from the MSN surface, which triggers the release of CPT and tolbutamide. Without glucose, drug leakage was minimal (10%–20%) over 6–12 h, but the release rate significantly increased with higher glucose concentrations. The cytotoxicity of the CPT‐loaded nanosystem was investigated, showing more than 75% cell viability. However, when the system was modified with FA, cell viability dropped to 40% at the same CPT concentration, but rose to 70% in a glucose‐free environment, indicating significant cytotoxicity in the presence of glucose [83].

Collectively, glucose‐responsive MSNs are most convincing when glucose consumption contributes directly to therapy. GOx‐based systems can combine starvation therapy, H_2_O_2_ generation, acidity amplification, and chemotherapy. However, glucose itself is not tumor‐specific; therefore, targeting or additional responsive mechanisms are usually required.

*ATP‐responsive MSNs*: ATP‐responsive MSNs exploit the concentration differences between healthy tissues and tumor cells. ATP is present at relatively low levels in healthy extracellular environments, reported to be around 1–5 μM, but can reach much higher levels in tumor cells, often over 100 μM, because of altered metabolism and excessive glycolysis [114]. This difference supports the ATP‐triggered drug release after cellular uptake. Common ATP‐recognition modules include ATP‐binding ssDNA aptamers and coordination‐based gatekeepers, which can be displaced by competitive ATP binding [115, 116]. However, since ATP is a universal intracellular molecule rather than a tumor‐exclusive marker, ATP‐responsive MSNs should be combined with tumor‐targeting or endocytosis‐dependent delivery.

Lai et al. developed an ATP‐responsive drug delivery system using upconversion nanoparticles (UCNP)‐based MSNs. This system was designed for real‐time monitoring and tracking of drug release in response to ATP. The bio‐gatekeeper consisted of a zinc‐dipicolylamine analog (TDPA‐Zn^2+^) immobilized on the nanoparticle's surface, which provided binding sites for branched polypeptides with carboxylate side chains. In the presence of ATP, these polypeptides were competitively displaced by ATP molecules, leading to the controlled release of the encapsulated drugs. The system demonstrated negligible leakage without ATP and concentration‐dependent release in the presence of ATP. At 5 mM ATP, DOX and CPT release reached approximately 85% and 64%, respectively, after 24 h [84]. He et al. designed another promising ATP‐responsive nanosystem using aptamer‐functionalized MSNs as a gate. In this system, the ATP aptamer was combined with two single‐stranded DNA arms (ssDNA1 and ssDNA2) to form a sandwich‐like DNA structure that blocked the MSNs' pores and prevented drug release. Upon encountering ATP, the aptamer bound ATP and dissociated from the arm DNA, opening the pore and allowing cargo release. This system achieved 83.2% release of the encapsulated cargo, demonstrating the flexibility and effectiveness of using ssDNA aptamers in ATP‐responsive drug delivery [85].

Overall, ATP‐responsive MSNs are suitable for intracellular release because ATP levels are much higher inside cells than in extracellular fluids. Their advantage is the availability of aptamer‐ or coordination‐based gates with good molecular recognition. Since ATP is universal in living cells, these systems should be paired with tumor targeting or uptake‐dependent delivery.

*Ionic strength‐responsive MSNs*: Studies have shown that sodium‐magnetic resonance imaging (^23^Na‐MRI) performed in breast cancer patients demonstrated an increased sodium content of up to 50%–70% in breast cancers compared to the healthy tissue milieu [117]. Excessive NaCl concentration (0.15 M) with interleukin‐17 (IL‐17) (0.1 nM) synergistically promotes breast cancer growth by provoking reactive nitrogen and oxygen (RNS/ROS) species, which also promote cancer [118]. To exploit this property for on‐demand cargo delivery, Zhu et al. tailored sodium polystyrene sulfonate (PSS) and the poly(allylamine hydrochloride) (PAH)‐gated ibuprofen‐loaded MSNs. At a higher salt concentration (10 mM), the high ionic strength reduced the electrostatic binding between the oppositely charged layers, which resulted in less compact PAH/PSS multilayers. The multilayers could no longer cap the mesoporous channels' openings and provide the pathways for drugs to spread into the release medium (∼80%) for 48 h. In contrast, at a low salt concentration (0.5 mM), only approximately 13% of the cargo leaked within the same period, encouraging the prospect of employing such a strategy for cancer treatment for future directions [86].

In general, ionic‐strength‐responsive MSNs demonstrate how electrostatic multilayer gates can regulate release through salt‐dependent changes. However, ionic strength is tightly controlled in vivo and is not a strong tumor‐specific trigger. Therefore, this strategy is better considered as a proof‐of‐concept or auxiliary gate rather than a stand‐alone cancer‐selective mechanism.

*Urea‐responsive MSNs*: Urea concentrations in tumors are 26% higher than those in plasma, owing to the dysregulation of the urea cycle [119]. The sensitivity of the urea‐responsive nanosystem depends on the transformation of urea to NH_4_HCO_3_ and NH_4_OH by the action of urease, which results in pH increase, which allows specific polymers that are subjected at high pH to be exposed to polymer erosion, which results in controlled drug release of loaded cargos [120]. Alternatively, urea facilitates the internalization of the NPs by endowing them with energy which acts as biofuel urea [121]. For example, Hortelao et al. reported urease‐mediated MSNs functionalized with PEG and anti‐FGFR‐3 antibodies to actively target bladder cancer. NPs motion is assisted by urea, which served as a fuel to allow NPs to swim in simulated and real urine, known as nanomotors or nanoswimmers. When urea is present, the internalization of NPs was remarkably higher than NPs in the absence of urea, making them an excellent design for active targeting and motion capabilities, which is 14 times higher than the passive targeting nanosystem without antibodies modification [122]. The same group reported urease‐powered DOX‐loaded MSNs, the urease enzyme coat allowed to exploit chemical energy and transform it into a mechanical movement for active transportation toward tumor cells. The presence of urea increased DOX release by approximately 4‐fold compared with passive nanobots. The release is attributed to the increased NPs' diffusion in urea and the flow generated on the surface. They also evaluated the drug release in the presence and absence of urea at different pH values (5, 6, 9, and 10) to confirm whether the change in pH caused by urease activity could affect the DOX release. As a result, the DOX release profile indicated that the release behavior was responsive to the presence of urea, regardless of the pH change of the medium [87]. Collectively, urea‐responsive MSNs are a specialized strategy in which urease can use urea to promote motion, local chemical change, or cargo release. Their limitation is that urea is not strongly tumor‐specific in most tissues. These systems are most suitable for urinary tract‐related applications or enzyme‐powered nanomotor designs.

Together, intrinsic stimuli‐responsive MSNs are attractive because they use endogenous biochemical differences between tumor and normal tissues. However, no intrinsic trigger is universally present or completely tumor‐specific. Therefore, the responsive mechanism should be matched to the dominant biological feature of the selected tumor type.

### 4.2 Extrinsic stimuli‐responsive MSNs

**FIGURE 5:**
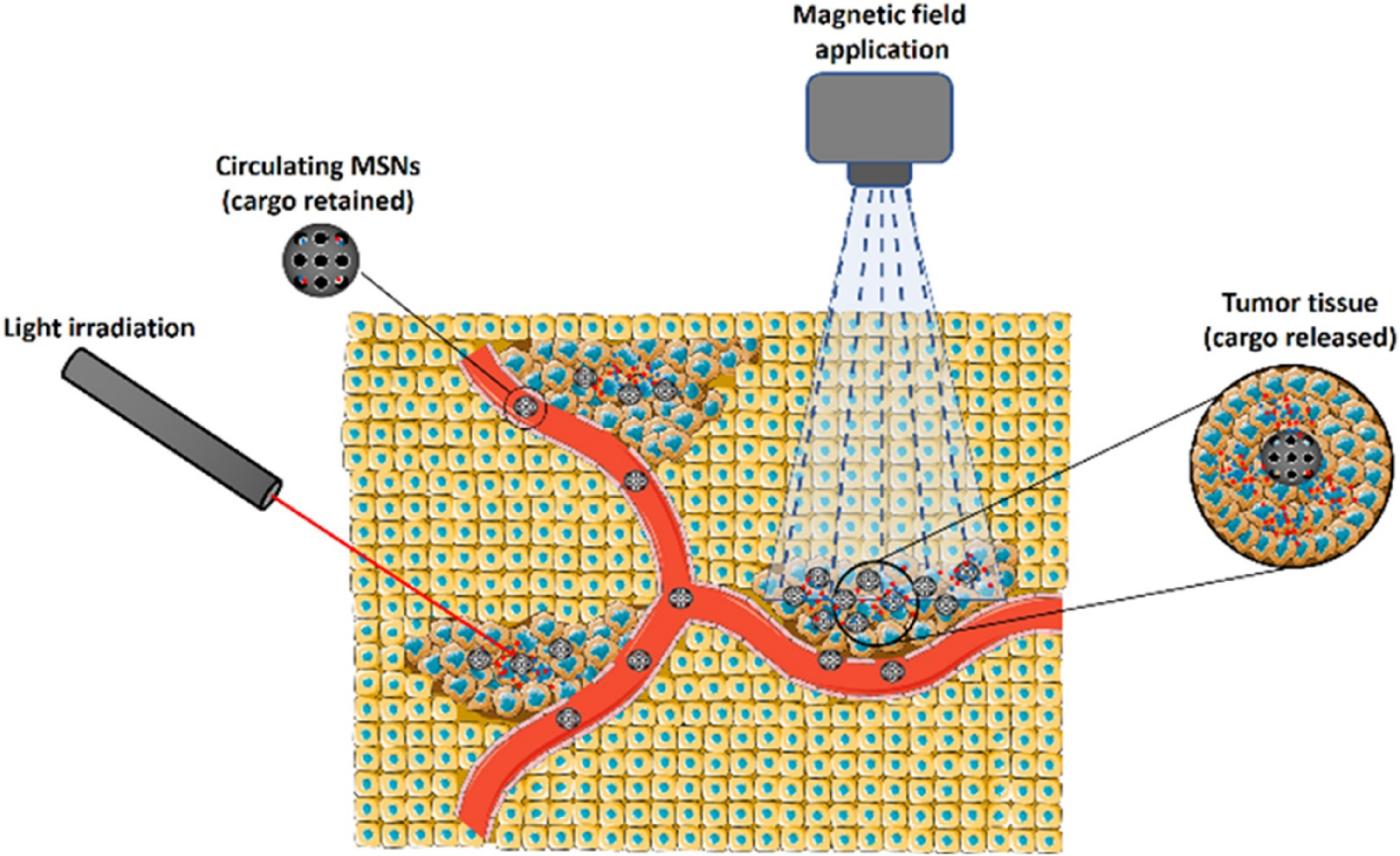
Schematic illustration of external stimuli applied to tumors to trigger cargo release from MSNs.

Unlike internal stimuli, external stimuli can be accomplished via an external physical treatment. External stimuli‐responsive NPs might be more favorable because of the precise spatiotemporal treatment and different physiological conditions of the human population [123]. In this section, the on‐demand release of cargo via the application of external stimuli (Figure 5), involving temperature variations, magnetic fields, ultrasound, light, and electric fields, are discussed.

*Light‐responsive MSNs*: Light‐responsive systems are attractive because they offer non‐contact activation and high spatial and temporal control over drug release. Light can be precisely applied to internal tumor areas using optical probes, making it a powerful tool for targeted cancer therapy [124]. Among various types of light, near‐infrared (NIR) light is particularly advantageous for clinical use because of its greater tissue penetration than UV or visible light, making it more favorable for targeting tumors [125]. Photodynamic therapy (PDT) and photothermal therapy (PTT) are two prominent light‐responsive strategies. PDT involves photosensitizers, such as chlorins and porphyrin derivatives, which generate cytotoxic singlet oxygen (^1^O_2_) and free radicals upon light irradiation to kill tumor cells [126]. In contrast, PTT utilizes nanoparticles at tumor sites to convert light into heat, thereby inducing cancer cell death [127, 128].

Recent advances in cancer phototherapy emphasize that PDT, PTT, and photoimmunotherapy act through ROS generation, heat production, and immune activation, respectively, but their clinical performance still depends on improving tumor targeting, photothermal conversion, ROS generation, and agent pharmacokinetics [129]. Initial studies demonstrated the use of light‐sensitive MSNs with coumarin molecules on the pore outlets, which underwent reversible dimerization in response to light, effectively controlling drug release [130]. However, the use of visible and ultraviolet (UV) light is limited due to low tissue penetration and potential damage to biological tissues. Luo et al. developed an indicator‐guided photo‐responsive MSN/Au system that combined MMP‐2 sensing with light‐triggered DOX release. MMP‐2‐mediated peptide cleavage restored fluorescence to indicate the tumor‐associated signal, after which UV irradiation induced azobenzene isomerization and opened the cyclodextrin‐based gate to release DOX. The system showed selective signal activation and enhanced cytotoxicity in SCC‐7 cells. Although UV irradiation limits deep‐tissue translation, this work illustrates how biological sensing can be used to guide the timing and location of externally triggered drug release [131].

In contrast, NIR light (650–900 nm) minimizes light scattering and absorption, allowing for deeper tissue penetration, which makes it a promising candidate for in vivo applications of photo‐controlled drug delivery [132]. Kumar et al. successfully integrated PDT, PTT, and chemotherapy into a single nanoplatform. This system utilized NaYF_4_/Tm/Er/Fe UCNP as a core, with a shell of NaYbF4:1% Tm. TiO_2_ nanoparticles and photocleavable Ru(II) complexes were decorated on the surface, and gold nanorods (GNRs) were attached for light‐responsive drug release. The system demonstrated a significant release of DOX (83% over 8 h) under NIR laser irradiation, confirming the effectiveness of the photochemical uncapping mechanism [133]. Carmona et al. presented a novel light‐responsive MSN system that released its cargo upon exposure to visible light from a standard lamp. The MSNs were loaded with a photosensitive compound and modified with porphyrin, which acted as a gatekeeper. Upon light exposure, the system released most of its cargo within 18 h, with negligible leakage in the absence of light [134]. Shi et al. developed a mitochondria‐targeted light‐responsive system using indocyanine green (ICG) as a photosensitizer. This system combined PDT and PTT by co‐loading ICG and DOX into MSNs, which were further modified with phase transition material L‐menthol (LM). The system showed rapid DOX release at elevated temperatures (45°C) and upon NIR irradiation, demonstrating the potential for controlled drug release triggered by photo‐hyperthermia [135]. Li et al. constructed NIR laser‐responsive MSNs coated with carbon nanotubes (CNTs) and modified with isobutyramide (IBAM) and human serum albumin as gatekeepers. The CNTs converted light to heat upon laser irradiation, disrupting the IBAM linker and enabling DOX release. This system showed a synergistic effect, enhancing the antitumor response through the combined photothermal action of CNTs and the chemotherapeutic action of DOX [136].

Overall, light‐responsive MSNs provide excellent spatial and temporal control and are well suited for PDT, PTT, and photo‐triggered release. Their major limitations are wavelength‐dependent tissue penetration and the potential for phototoxicity or thermal damage at excessive irradiation doses. They are therefore most suitable for superficial tumors or lesions accessible by intraoperative, endoscopic, or image‐guided irradiation.

*Magnetic‐responsive MSNs*: Magnetic‐responsive drug delivery systems utilize an external magnetic field to guide magnetic nanoparticles (mNPs) to the target location within the body. These nanoparticles typically contain magnetic elements such as iron, manganese, nickel, cobalt, chromium, gadolinium, and their derivatives. Among them, magnetite (Fe_3_O_4_) and maghemite (γ‐Fe_2_O_3_) are the most commonly used in biomedical applications [137].

In these systems, on‐demand drug release can be triggered by applying a magnetic field. The high‐frequency magnetic field causes the mNPs encapsulated within the MSN matrix to rotate generating heat. This heat can increase the pore volume from 0.067 to 0.127 cm^3^/g, facilitating drug release. Additionally, magnetic field‐induced vibrations can actively pump the loaded drug out of the pores [138]. When combined with temperature‐responsive gate‐coated MSNs, the escalating temperature and vibrational effects synergistically enhance drug release upon magnetic field irradiation.

However, one of the challenges with magnetic guidance is overcoming the high blood flow rate (greater than 10 cm/s in arteries and 0.05 cm/s in capillaries), which can hinder the movement of the magnetic platforms upon intravenous administration. Therefore, the development of powerful magnets capable of generating strong magnetic fields to effectively manipulate particles is essential [139]. Chen et al. developed a promising nanosystem in which CPT‐loaded MSNs were capped with monodispersed Fe_3_O_4_ nanoparticles. The chemical bonding between the Fe_3_O_4_ NPs and MSNs provided strong adhesion, effectively capping the pores and reducing drug leakage to 2.6%, compared to 95% leakage in non‐capped MSNs. This system also demonstrated enhanced capabilities for MRI and fluorescence imaging while controlling drug release upon magnetic field irradiation. Upon 5 min of magnetic field exposure, 21.9% of CPT was released within the first 10 min, with a cumulative release rate of 45.9% within 24 h. In vitro anticancer activity showed a significant reduction in cancer cell viability, with 42% cell death upon magnetic field stimulation, compared to over 80% viability without the stimulus [140]. Another example is the work by Guisasola et al., who prepared superparamagnetic iron oxide nanoparticles (SPIONs)‐embedded MSNs. Upon applying an alternating magnetic field (AMF), the magnetic nanoparticles converted the AMF into heat, which destabilized the temperature‐responsive polymer gatekeeper on the MSNs' surface, leading to drug release. This system achieved robust tumor growth inhibition through the synergistic effects of hyperthermia and chemotherapy with DOX [141]. Chen et al. developed a magnetic MSN platform integrating magnetic targeting, T2‐weighted MRI, RGD‐mediated tumor targeting, and redox‐responsive drug release. A reduction‐sensitive Pt(IV) linker anchored the β‐cyclodextrin gatekeeper, enabling intracellular reductive conditions to trigger DOX release while activating Pt(II) chemotherapy. Magnetic assistance further enhanced tumor accumulation and therapeutic efficacy. This study illustrates how magnetic guidance and imaging can be combined with biologically triggered intracellular release rather than relying solely on magnetic actuation [142].

Collectively, magnetic‐responsive MSNs can integrate magnetic targeting, MRI, hyperthermia, and triggered release. However, magnetic guidance becomes less efficient than the target depth and distance from the external magnet increase, while rapid blood flow can further limit particle retention. Field strength, magnetic‐field gradient, and heating conditions must also remain within clinically acceptable ranges. These systems are therefore most attractive when magnetic guidance, imaging, or hyperthermia provides a clear therapeutic advantage.

*Ultrasound‐responsive MSNs*: Ultrasound (US) is a noninvasive and straightforward tool that enables precise tumor targeting and drug release while limiting exposure of surrounding healthy tissues [143]. Typically generated by piezoelectric transducers, US converts electrical signals into mechanical waves that travel through fluids [144]. However, in clinical settings, US waves have limited scattering in the blood at frequencies between 1 and 40 MHz, which can pose challenges for imaging. To enhance the contrast in US imaging, researchers have developed contrast agents containing gas encapsulated in biodegradable shells [145]. Microbubbles (MBs) are particularly effective as contrast agents and are widely used for tissue perfusion and delineation in US imaging. They also serve as vehicles for drug delivery, allowing remote‐controlled, on‐demand drug release, and enhanced intracellular drug accumulation via MB bursting upon exposure to high‐intensity US [146].

When MBs are subjected to high‐intensity US (>1 MPa, 1 MHz), they undergo rapid oscillations that lead to transient cavitation, producing microjets and shockwaves. These microjets, powerful streams of liquid generated by the asymmetric collapse of MBs, can create transient or transiently increased vascular and cell‐membrane permeability, thereby enhancing the extravascular delivery of drugs to targeted sites [147]. Despite its potential, US‐triggered therapy faces challenges, particularly in designing nanoparticles that are both biologically active and sensitive to US [148]. Jin et al. addressed this challenge by proposing the generation of MBs from interfacial nanobubbles (INBs) using clinically available transducers. They constructed superhydrophobic MSNs capable of adsorbing INBs within their mesopores and on their surfaces. These bubble precursors remained stable until they were transformed into MBs under sufficient acoustic pressure, allowing controlled US‐triggered drug release [149]. In another study, Lv et al. developed a FA‐modified MSN system loaded with tanshinone IIA (TAN) and encapsulated in MBs, termed MSNs‐FA‐TAN‐MBs. Upon reaching the target site, US irradiation disrupted the microbubbles, releasing the cargo on demand. This approach demonstrated dose‐dependent inhibition of HeLa and A549 cancer cell growth, with MSNs‐FA‐TAN showing significantly higher cytotoxicity than free TAN or TAN‐loaded MSNs [150]. Wang et al. synthesized a PTX‐loaded FA‐β‐CD‐capped MSN system, which was designed to store gas within the internal hydrophobic channels of MSNs, serving as an ultrasonic cavitation nucleus. The final product, PTX@FA‐β‐CD/H‐MSNs, exhibited improved cavitation effects and achieved rapid on‐demand drug release upon US application. The cumulative release of PTX increased proportionally with US intensity, and the tumor growth inhibition rate in mice was concentration‐dependent in the presence of US [151]. Du et al. further advanced this field by developing US‐responsive magnetic MSNs (mMSNs)‐loaded lipid microbubbles (M‐MSNs@MBs) for US‐mediated imaging and gene transfection. In this system, plasmid DNA was loaded into the mesopores, and the NPs were guided to the cancer site using a magnetic field to achieve targeted therapy. Upon US application, the cargo was released and cell‐membrane permeability was enhanced, improving NP penetration through tumor tissues. This system demonstrated a promising platform for noninvasive gene therapy, with the highest gene transfection efficiency observed when US and M‐MSNs@MBs were combined [152].

In general, ultrasound‐responsive MSNs are attractive for deeper tumors because ultrasound is clinically established and penetrates tissue better than light. Their limitations include dependence on acoustic parameters, cavitation behavior, and microbubble stability, which can affect the reproducibility of triggered release. They are most suitable when noninvasive deep‐tissue activation is required.

*Temperature‐responsive MSNs*: Temperature‐responsive MSNs offer a versatile approach for controlled drug delivery, particularly in cancer therapy, by leveraging both endogenous and external temperature stimuli. These nanosystems are designed to release their cargo in response to elevated temperatures, which can be induced by external triggers such as hyperthermia in the targeted tissues. This strategy not only enables the precise release of drugs but also enhances the effectiveness of thermal‐based therapies when combined with chemotherapy [67].

Hyperthermia treatment for tumors involves the application of heat to tumor tissues to inhibit cellular proliferation, regulate cell growth, and induce apoptosis. Magnetic hyperthermia, which uses mNPs to generate localized heat, is a promising biomedical application in cancer therapy [153]. Thermoresponsive polymers play a crucial role in these systems, undergoing a transition from a coil to a globule structure at their lower critical solution temperature (LCST). Poly(N‐isopropylacrylamide) (PNIPAAm) is one of the most studied thermoresponsive polymers, with an LCST around 30–32°C, making it particularly interesting for drug delivery applications [154]. Thermoresponsive polymers, such as PNIPAAm, undergo a coil‐to‐globule transition around their LCST, enabling temperature‐triggered pore opening or shell collapse. However, pure PNIPAAm often requires copolymerization or formulation adjustment to align its LCST with clinically useful hyperthermia ranges [155].

For instance, Peralta et al. developed magnetic and temperature‐responsive nanoparticles by conjugating PNIPAAm with 3‐(methacryloxypropyl)trimethoxy silane (MPS) onto mMSNs to create a core‐shell structure (mMSNs‐PNIPAAm‐co‐MPS). This design enabled on‐demand drug release, with 100% of the cargo released at 40°C within 16 h—five times higher than the control at lower temperatures. The study demonstrated the potential of mMSNs‐PNIPAAm‐co‐MPS as a promising candidate for stimuli‐responsive drug delivery systems, particularly for cancer treatment where complete cargo release can be achieved through heat irradiation [156]. Another approach by Singh et al. involved a core‐shell design using an aqueous free radical polymerization technique. They covalently cross‐linked a PEG‐based polymer shell to the MSN surface, which provided extra stability and reduced drug leakage at temperatures below the LCST (37°C). This design significantly improved the release rate, achieving approximately 85% release when the temperature was above the LCST, demonstrating the effectiveness of this method in controlled drug delivery [157].

Overall, temperature‐responsive MSNs are useful when drug release can be coupled with controlled hyperthermia. Their advantages include simple heat‐triggered gate opening or polymer collapse. However, the transition temperature must be carefully tuned to prevent premature release at body temperature, while local heating should be sufficiently controlled to avoid damage to surrounding tissues. These systems are therefore most suitable when localized and monitorable hyperthermia can be achieved.

*Electro‐responsive MSNs*: Electro‐responsive MSNs enable externally controllable and on‐demand drug release through applied electrical stimulation, which can induce electrochemical redox reactions or the movement of charged molecules [23]. This method leverages the ability of certain molecules to align with an applied electric field, enabling controlled on‐demand drug release. Most electro‐responsive systems rely on intrinsically conducting polymers (ICPs), such as polypyrrole (PPy) [158] and polyaniline (PANI) [159], or materials containing electrically‐polarizable particles suspended in an insulating liquid, such as silicone or mineral oil, which exhibit electrically‐tunable viscoelasticity [160].

Since MSNs are not intrinsically electro‐responsive, most reported electro‐responsive systems rely on conductive polymers or electrically active coatings to translate electrical stimulation into pore opening, gate cleavage, or molecular movement. Typical examples include PPy‐, PANI‐, and poly(3,4‐ethylenedioxythiophene) (PEDOT)‐based MSN composites [161, 162].

For example, Cheng et al. reported the first PPy–SBA‐15 nanocomposites, utilizing SBA‐15 for its larger mesopores and thicker walls compared to MCM‐41. The pyrrole monomer was polymerized within the channels of SBA‐15, preventing the formation of PPy layers on the nanoparticle's outer surface [163]. Similarly, Fang et al. created PPy‐functionalized MCM‐41 composites with enlarged pore diameters by modifying the synthesis process, demonstrating improved responsiveness [164]. More recently, Fernandez et al. advanced the electro‐responsive nanomedicine field by developing an RhB‐loaded nanosystem based on conductive polymer‐gated MSNs. The system employed a poly(3,4‐ethylenedioxythiophene)‐doped polymer, with bipyridinium cations and heparin acting as gatekeepers. Cargo release was triggered by an applied electrical potential, which reduced the bipyridinium groups and weakened their electrostatic interaction with the heparin cap. Heparin detachment opened the pores and enabled near‐complete RhB release within 1 h. When the conductive polymer was covalently attached to the MSN surface, it significantly reduced cargo leakage (less than 5%) and achieved near‐complete drug release within 60 min under electrostatic potential. This platform demonstrated excellent biocompatibility and effective on‐demand drug release in HeLa adenocarcinoma cells [165]. Collectively, electro‐responsive MSNs can provide rapid release under an applied electrical stimulus. Their main limitation is the need for local electrical access, electrodes, or implantable devices. Therefore, they are more suitable for localized or device‐assisted therapy than systemic treatment of deep tumors.

Taken together, extrinsic stimuli‐responsive MSNs provide stronger spatial and temporal control than purely endogenous‐responsive systems, but their clinical utility depends on tumor depth and accessibility, tissue penetration of the applied energy, device availability, treatment safety, and the reproducibility of stimulus delivery. The choice of an external trigger should therefore be guided by whether it provides a clear therapeutic or imaging advantage that justifies the additional device and formulation complexity.

## 5 MULTI‐STIMULI‐RESPONSIVE MSNS AND COMBINATION THERAPEUTIC SYSTEMS

Single‐stimulus MSNs work well when one trigger is dominant, reliable, and clearly different between tumor and normal tissue. However, tumors are heterogeneous. Acidic pH, enzyme overexpression, redox gradients, ROS, hypoxia, and metabolic changes differ by tumor type, disease stage, vascular region, and intracellular compartment. Therefore, multi‐stimuli‐responsive MSNs have been developed to improve specificity, reduce premature leakage, and enable sequential or synchronized therapy [10, 166].

### 5.1 Dual‐ and multi‐input logic‐gated MSNs

The purpose of multi‐stimuli design is not simply to add more functions. It is to improve release selectivity. In a logic‐gated system, one stimulus may prime or localize the carrier, while another opens the gate or amplifies therapy. Multi‐stimuli‐responsive systems can generally follow either sequential or synergistic activation modes. In a sequential design, the first stimulus may promote tumor accumulation, cellular uptake, or partial gate opening, whereas the second stimulus subsequently triggers cargo release or activates an additional therapeutic modality. In contrast, synergistic systems rely on the concurrent or mutually reinforcing effects of multiple stimuli to enhance drug release or therapeutic efficacy. Therefore, the order, timing, and intensity of each stimulus should be matched to the intended biological function rather than simply combining multiple responsive elements within the same carrier. Dual‐responsive MSNs are often the most practical because they improve selectivity while keeping synthesis and characterization manageable. Enzyme/redox‐responsive MSNs based on dopamine, peptide bonds, and disulfide bonds released cargo in response to GSH or pepsin [167]. GSH/α‐amylase‐responsive starch‐conjugated MSNs and hypoxia/redox‐responsive MSNs demonstrate the combination of two biological cues [168]. pH/temperature‐responsive MSNs respond to acidic and thermal conditions from tumors or hyperthermia‐treated tissues [169].

**FIGURE 6:**
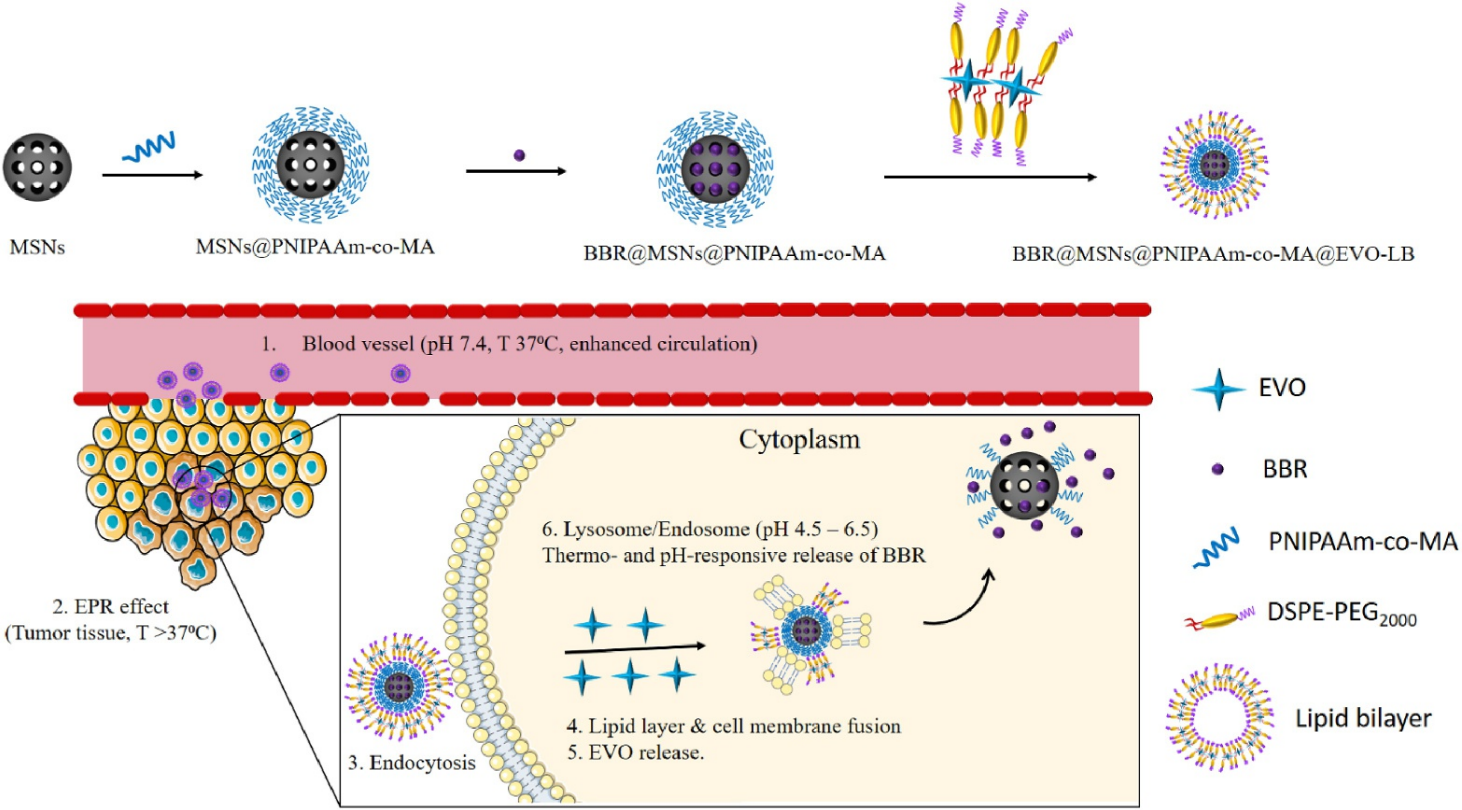
Schematic illustration of pH‐ and thermoresponsive MSNs for a drug‐delivery nanosystem.

Triple‐ and quadruple‐responsive MSNs can provide more elaborate control, but each additional component increases synthesis complexity, characterization burden, batch variability, and regulatory uncertainty. Magnetic/redox/temperature‐responsive MSNs achieved enhanced DOX release under elevated temperature, reducing conditions, and magnetic fields [170]. pH/temperature/redox‐responsive polymers also showed multi‐cue release behavior (Figure 6) [171]. Quadruple‐responsive systems integrating pH, temperature, NIR light, and ethylenediamine responsiveness produced synergistic chemo‐photothermal therapy [172]. These examples are useful, but the key question is whether each trigger addresses a defined biological or therapeutic problem.

Thus, dual‐input systems are often more practical than highly complex multi‐input systems. Additional triggers should be included only when each plays a clear role, such as improving accumulation, opening the gate, or amplifying therapy.

### 5.2 Chemotherapy combined with phototherapy or hyperthermia

Multi‐stimuli‐responsive MSNs are well‐suited for combination therapy. Chemotherapeutics can be loaded into mesoporous channels, whereas photosensitizers, photothermal agents, magnetic nanoparticles, and thermoresponsive polymers can be integrated into the same carrier. Intrinsic tumor cues can reduce leakage and promote intracellular release, whereas light, magnetic fields, ultrasound, and heat provide spatially controlled activation [123, 157]. This arrangement supports chemotherapy combined with photodynamic therapy, photothermal therapy, magnetic hyperthermia, ultrasound‐mediated cavitation, and thermal release. The most compelling systems are those in which each component contributes to a single treatment workflow rather than adding unrelated functions. Luo et al. reported a coordinated multi‐stimuli MSN platform based on mesoporous silica‐coated gold nanorods for combined chemotherapy, PDT, and PTT. The gold nanorod core acted as a photothermal transducer, the mesoporous silica shell carried AlPcS4, and β‐cyclodextrin was attached through a redox‐cleavable Pt(IV) linker to provide GSH‐responsive gating. Under reducing conditions, approximately 58% of AlPcS4 was released within 24 h, and NIR irradiation further accelerated the release. In HepG2 cells, combined irradiation at 808 and 660 nm reduced cell survival to approximately 8%, substantially lower than either single‐laser treatment. In vivo, fluorescence imaging was used to select the irradiation time, and the dual‐laser treatment produced the strongest tumor inhibition. This study illustrates how redox‐triggered release, chemotherapy, PDT, PTT, targeting, and imaging can be integrated into a temporally coordinated therapeutic sequence [173].

Taken together, combination MSN systems are strongest when chemotherapy, phototherapy, and hyperthermia work in a coordinated sequence. Their benefits should be proven against simpler single‐stimulus or single‐therapy controls.

### 5.3 Imaging‐guided and theranostic MSN platforms

Imaging‐guided MSNs include fluorescent dyes, magnetic components, UCNPs, gold nanostructures, gadolinium complexes, and related modules to track the distribution, release, and treatment response. Imaging is especially useful before external activation because it can confirm tumor localization before light, magnetic‐field, ultrasound, or heat stimulation is applied. Simultaneously, theranostic designs should be used only when they add practical functions. Imaging modules not only improve treatment precision but also increase formulation complexity. Their use should be justified by functions such as guiding irradiation, confirming accumulation, monitoring the release, and assessing the response.

In summary, imaging modules should guide real therapeutic decisions, such as confirming tumor accumulation, choosing the irradiation time, monitoring release, or assessing early response. Otherwise, they may add complexity without improving treatment value.

### 5.4 Design principles for synchronized cargo release

Multi‐stimuli‐responsive MSNs should be designed with several constraints in mind. The selected stimuli should fit the tumor type and administration route. The response threshold should match physiologically achievable conditions rather than extreme in vitro settings [174]. The carrier should remain stable during storage and circulation but respond after tumor accumulation or following external activation. The number of functional modules should be kept as low as possible while preserving the therapeutic performance. Finally, any benefit should be tested against simpler single‐stimulus or non‐responsive controls. In this context, smartness refers to the useful integration of tumor biology, gatekeeping chemistry, cargo properties, activation modality, and degradation behavior, not the largest possible number of triggers.

The sequence and compatibility of different functional modules should also be considered. In sequential systems, activation of one module should not compromise the function of another module that is required at a later stage. For example, heat or ROS generated by photothermal or photodynamic components should not prematurely damage or inactivate targeting ligands, responsive gatekeepers, or sensitive cargos before sufficient tumor accumulation has occurred. Similarly, premature opening of one gate may alter the local microenvironment or release kinetics and thereby affect the response of a second module. Therefore, the timing, intensity, and order of each stimulus should be matched to its intended biological or therapeutic role.

Appropriate controls are also necessary to demonstrate whether the added complexity provides a meaningful benefit. Single‐stimulus, non‐responsive, non‐targeted, and free‐drug controls can help distinguish the contribution of individual modules and determine whether the intended sequential activation or genuine synergistic benefit has been achieved.

## 6 OUTLOOK AND FUTURE PERSPECTIVES

### 6.1 Disease‐driven responsive design

The next stage of MSN design should move beyond adding responsive modules to one nanoparticle. Multi‐stimuli systems can improve selectivity and coordinate therapy; however, more triggers do not automatically mean better treatment. Each gatekeeper, linker, coating, ligand, or external‐activation component can increase synthetic complexity, lower loading capacity, reduce reproducibility, and complicate quality control.

The design should start from the disease setting. Tumor location, vascularization, dominant microenvironmental cues, penetration barriers, feasible external triggers, cargo types, and administration routes should be considered when determining the responsive module. For example, magnetic or ultrasound activation may be more relevant than UV or visible light for deep‐seated tumors; urea‐powered systems may be particularly suitable for bladder or other urinary tract tumors where urinary urea is directly accessible; and hypoxia‐responsive systems are more rational for tumors or tumor regions with pronounced hypoxia, although poor vascularization may require improved penetration and distribution before additional gatekeepers become useful. Immunotherapeutic MSNs should be optimized for antigen‐presenting cell uptake and immune activation, rather than solely for tumor cell cytotoxicity. In this sense, a smart MSN is one that solves a defined biological or therapeutic problem.

### 6.2 Expanding stimuli‐responsive MSNs toward cancer immunotherapy

Stimuli‐responsive MSNs may also contribute to cancer immunotherapy. Nanomedicine is moving toward actively targeted, stimuli‐responsive, combination, and immunotherapy‐oriented formulations [1, 2]. Several MSN systems point in this direction. Extra‐large‐pore hollow MSNs can load cancer vaccines, and polyethylenimine (PEI) coating can provide an adjuvant‐like function [175]. Ce6/CpG‐modified MSNs can combine PDT with immune activation [80]. R848‐loaded MSNs can accumulate in migratory dendritic cells and induce T‐cell proliferation [176]. Virus‐like HMSNs loaded with DOX can promote tumor cell death and generate debris that is taken up by dendritic cells to activate T cells [177].

Future immunotherapeutic MSNs may deliver adjuvants, tumor antigens, nucleic acids, immune agonists, or immunoregulatory molecules with controlled timing and localization. Photothermal therapy, photodynamic therapy, ultrasound, and magnetic hyperthermia may amplify immune responses by increasing tumor cell damage, antigen exposure, inflammatory signaling, and immune cell recruitment. However, immune outcomes depend on cargo selection, particle size, surface charge, release kinetics, administration route, degradation behavior, and baseline tumor immunity. Dendritic cell maturation, antigen presentation, T‐cell activation, cytokine profiles, immune memory, and long‐term immune safety need systematic evaluation.

### 6.3 Degradable MSNs and long‐term biosafety

Long‐term safety remains a major barrier to MSN use. Although some studies have reported low toxicity at pharmacologically relevant concentrations, biocompatibility, biodistribution, degradation, and clearance depend on mesoporosity, size, shape, surface chemistry, cargo, and dose [178]. Ultrasmall silica nanoparticles, such as Cornell dots, have been used in first‐in‐human cancer imaging studies, and synthetic amorphous silica has been approved for food‐related uses at specified levels [179]. These findings are encouraging; however, they cannot be generalized to all MSN formulations.

The stable Si‐O‐Si framework may degrade slowly in vivo. Incomplete degradation can lead to retention, aggregation, organ accumulation, inflammation, and delayed toxicity. Clearance is influenced by particle size, PEGylation, and morphology; smaller silica nanoparticles may be excreted faster, PEGylation can reduce liver, lung, and spleen accumulation, and short rods may be cleared more efficiently than long rods [180]. Degradable MSNs and Mesoporous organosilica nanoparticles (MONs) can help address this issue. Degradation can be accelerated by incorporating metal oxides, organic/inorganic components, or cleavable bonds into the framework, including acid‐ or H_2_O_2_‐driven reactions and disulfide, acetal, Schiff base, or related labile groups [181]. Mesoporous organosilica nanoparticles retain the loading advantages of MSNs while improving biodegradability, and selected MON systems have shown gradual morphological disintegration without obvious pathology in the evaluated animal models [182].

### 6.4 Manufacturing, reproducibility, and clinical translation

Translation also requires attention to manufacturing and regulations. Many responsive MSNs use several synthetic steps, complex gatekeepers, ligand conjugation, hybrid organic‐inorganic structures, or external trigger‐responsive modules. Such designs are useful for proof‐of‐concept studies but can complicate scale‐up production, sterilization, batch reproducibility, storage stability, quality control, and regulatory review.

The key quality attributes include particle size distribution, pore size, morphology, surface charge, drug loading, encapsulation efficiency, gatekeeper density, ligand conjugation efficiency, residual surfactants or solvents, endotoxin level, colloidal stability, degradation profile, and release kinetics. Responsiveness itself should also be treated as a quality attribute, with quantitative testing of stimulus threshold, response rate, leakage under physiological conditions, release under biologically relevant conditions, and stability in serum‐containing media. Future studies should integrate pharmacokinetics, biodistribution, degradation, excretion, immune response, therapeutic efficacy, and organ toxicity into their research designs. Comparisons with clinically relevant controls are needed to determine whether a complex responsive MSN is superior to a simpler formulation.

From a translational perspective, responsive MSN systems should be judged less by how many modules they contain and more by whether they can be made reproducibly, keep the cargo inside during circulation, degrade in a controllable way, and respond to stimuli that can realistically be achieved in patients. These points should be evaluated together with pharmacokinetics, biodistribution, organ retention, immune response, and direct comparison with simpler formulations.

## 7 CONCLUSIONS

Smart MSNs provide a versatile platform for precision cancer therapies. Their value arises from the combination of an ordered mesoporous architecture, tunable physicochemical properties, surface functionalization, molecular gatekeeping, and controlled release. Intrinsic stimuli, such as pH, enzymes, redox potential, ROS, hypoxia, glucose, ATP, ionic strength, and urea, offer tumor‐associated biochemical triggers. External stimuli, such as light, magnetic fields, ultrasound, heat, and electric fields, provide controllable physical activation. Multi‐stimuli‐responsive systems can expand their therapeutic function when designed based on real tumor biology and feasible activation conditions.

Future studies should prioritize physiologically relevant responsiveness, simplified design, controllable biodegradation, reproducible manufacturing, and clinically feasible activation modalities. With a disease‐matched design and rigorous comparison against simpler controls, stimuli‐responsive MSNs may progress from proof‐of‐concept nanocarriers to practical smart systems for precision oncology.

## CONFLICT OF INTEREST STATEMENT

The authors declare no conflicts of interest.

## Data Availability

The data that support the findings of this study are available from the corresponding author upon reasonable request.
